# Lack of beta-arrestin signaling in the absence of active G proteins

**DOI:** 10.1038/s41467-017-02661-3

**Published:** 2018-01-23

**Authors:** Manuel Grundmann, Nicole Merten, Davide Malfacini, Asuka Inoue, Philip Preis, Katharina Simon, Nelly Rüttiger, Nicole Ziegler, Tobias Benkel, Nina Katharina Schmitt, Satoru Ishida, Ines Müller, Raphael Reher, Kouki Kawakami, Ayumi Inoue, Ulrike Rick, Toni Kühl, Diana Imhof, Junken Aoki, Gabriele M. König, Carsten Hoffmann, Jesus Gomeza, Jürgen Wess, Evi Kostenis

**Affiliations:** 1 Molecular, Cellular and Pharmacobiology Section, Institute for Pharmaceutical Biology, University of Bonn, Nussallee 6, 53115 Bonn, Germany; 2 Graduate School of Pharmaceutical Science, Tohoku University, Sendai, 980-8578 Japan; 3 PRESTO, Japan Science and Technology Agency (JST), 4-1-8, Honcho, Kawaguchi, 332-0012 Japan; 4 Institute for Molecular Cell Biology, CMB-Center for Molecular Biomedicine, University Hospital Jena, Hans-Knöll-Strasse2, 07745 Jena, Germany; 5 Bio-Imaging-Center/Rudolf-Virchow-Center, Institute of Pharmacology, University of Wuerzburg, Versbacher Str. 9, 97078 Würzburg, Germany; 6 Institute for Pharmaceutical Biology, University of Bonn, Nussallee 6, 53115 Bonn, Germany; 7 Pharmaceutical Biochemistry and Bioanalytics, Institute of Pharmacy, University of Bonn, An der Immenburg 4, 53121 Bonn, Germany; 8 AMED-CREST, Japan Agency for Medical Research and Development, 1-7-1 Otemachi, Tokyo, 100-0004 Japan; 9 Molecular Signaling Section, Laboratory of Bioorganic Chemistry, National Institute of Diabetes and Digestive and Kidney Diseases (NIDDK), Bethesda, MD 20892 USA

## Abstract

G protein-independent, arrestin-dependent signaling is a paradigm that broadens the signaling scope of G protein-coupled receptors (GPCRs) beyond G proteins for numerous biological processes. However, arrestin signaling in the collective absence of functional G proteins has never been demonstrated. Here we achieve a state of “zero functional G” at the cellular level using HEK293 cells depleted by CRISPR/Cas9 technology of the Gs/q/12 families of Gα proteins, along with pertussis toxin-mediated inactivation of Gi/o. Together with HEK293 cells lacking β-arrestins (“zero arrestin”), we systematically dissect G protein- from arrestin-driven signaling outcomes for a broad set of GPCRs. We use biochemical, biophysical, label-free whole-cell biosensing and ERK phosphorylation to identify four salient features for all receptors at “zero functional G”: arrestin recruitment and internalization, but—unexpectedly—complete failure to activate ERK and whole-cell responses. These findings change our understanding of how GPCRs function and in particular of how they activate ERK1/2.

## Abstract (web summary)

Arrestins terminate signaling from GPCRs, but several lines of evidence suggest that they are also able to transduce signals independently of G proteins. Here, the authors systematically ablate G proteins in cell lines, and show that arrestins are unable to act as genuine signal initiators.

## Introduction

About 20 heterotrimeric αβγ guanine nucleotide-binding proteins (G proteins) and 2 non-visual arrestins ensure signaling and regulation of several hundred G protein-coupled receptors (GPCRs), the largest family of membrane proteins in the mammalian genome^1^. By its very nature, this arrangement entails highly conserved mechanisms of activation, signal transduction and regulation. The prevailing view for long has been that GPCR signaling commences with activation of G proteins and is terminated by arrestins^2^. Arrestins, in particular β-arrestin 1 and 2 (βarr1/2, also known as arrestin2 and arrestin3, respectively), are recruited to activated GPCRs to which they bind tightly for two purposes: (i) arrest of further G protein signaling by steric hindrance, and (ii) removal of activated receptors from the cell surface by clathrin-dependent endocytosis. In this way, arrestins uncouple GPCRs from G protein pathways and desensitize the G protein-mediated response^3,4^.

During the past two decades, numerous reports have appeared to challenge the canonical ON-OFF paradigm. Functional outcomes downstream of activated GPCRs have been described that apparently do not require G protein participation but instead rely on β-arrestins as genuine signal initiators^5–11^. G protein-independent, arrestin-dependent signaling, or short “arrestin-dependent signaling” is a term widely used to denote this form of signal transduction and is now perceived by some as valid paradigm for the entire GPCR family^12^. Others refer to “arrestin-dependent mechanisms” with the implicit understanding that they are also G protein-dependent^13–17^. Hence, arrestin-dependent signaling mechanisms are an area in need of mechanistic and conceptual clarification.

We appreciate the large body of excellent experimental evidence addressing GPCR β-arrestin interaction up to atomic level resolution^17–19^ as well as the sophisticated biophysical studies resolving the fine details of arrestin conformational changes imparted by activated receptors^20,21^ and its functional consequences^22,23^. Despite these enormous advances in understanding the biophysical facets of arrestin function, the role of heterotrimeric G proteins and how they interplay with arrestin-mediated processes remains largely unclear, in part ascribed to the lack of tools for specific and quantitative elimination of all relevant G protein signaling routes.

Here we take advantage of human embryonic kidney cells (HEK293) depleted by CRISPR/Cas9 technology of either Gα proteins or arrestins^22,24–26^ along with selective G protein inhibitors^26^, wild-type, G protein-uncoupled and arrestin-uncoupled receptor variants as well as so-called “unbiased” and “arrestin-biased” ligands to visualize and isolate the independent signaling options. By creating two unambiguous experimental conditions, “zero functional G” vs. “zero arrestin”, we investigate using a panel of seven family A rhodopsin-like receptors from different coupling classes, (i) downstream signaling consequences and (ii) their upstream driving forces. We place particular emphasis on mechanisms underlying mitogenic signaling via the extracellular signal-regulated kinase 1/2 (ERK1/2) cascade, a fundamental signaling pathway controlling proliferation, differentiation, and survival of cells, and one of the earliest and most prominent examples for G protein-independent, arrestin-dependent signaling^7^. For this pathway, arrestin-dependence^12–17,27^ but not G protein-independence^7,28,29^ has been investigated extensively. Moreover, we attempt to visualize arrestin-driven signaling using label-free phenotypic whole-cell biosensing based on dynamic mass redistribution (DMR), a technology platform competent to portray a multitude of cellular events downstream of signaling-competent proteins^30–32^. We find that G proteins but—unexpectedly—not arrestins initiate ERK signaling and phenotypic cell morphology changes in their own right. These data change our perception of how GPCRs signal cells and emphasize the vital role of G proteins rather than arrestins as genuine drivers of GPCR-mediated signal transduction.

## Results

### GPCRs recruit arrestins in the absence of active G proteins

**Fig. 1:**
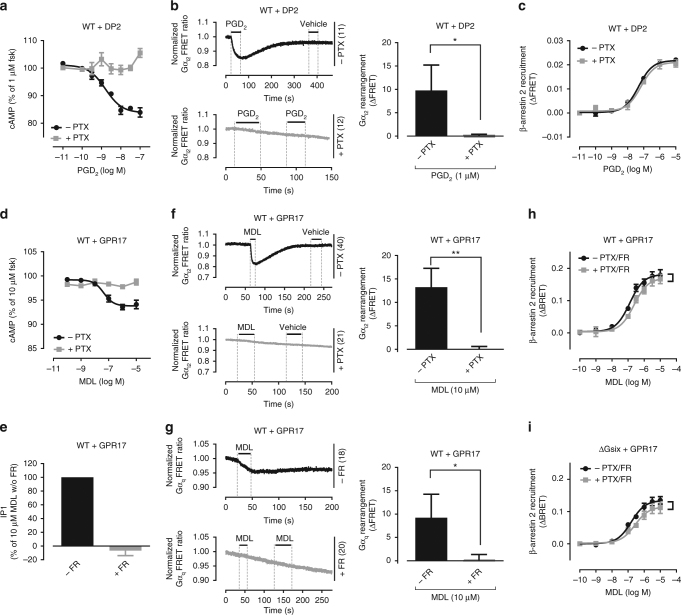
G protein activation and βarr2 recruitment in absence and presence of active G proteins. **a** PGD_2_-mediated depression of cAMP production in DP2-HEK293 cells, in the absence or presence of PTX. **b** Representative real-time FRET ratios and summary of Gi_2_ protein rearrangement after stimulation with PGD_2_ in DP2 expressing wild-type (WT) cells in absence and presence of PTX. **c** β-arrestin2 recruitment to PGD_2_-stimulated DP2 receptors, in the absence or presence of PTX. **d** Effect of MDL on the production of cAMP by Gi/q-linked GPR17, in the absence or presence of PTX. **e** IP1 accumulation of MDL-activated GPR17, in the presence or absence of FR. **f**, **g** Gi_2_ (**f**) and Gq (**g**) protein rearrangement in GPR17 expressing wild-type (WT) cells stimulated with MDL in absence and presence of G protein inhibitors. **h**, **i** β-arrestin2 recruitment to MDL-stimulated GPR17 when Gi/q is inhibited with PTX and FR (**h**) or when the entire complement of cellular Gα proteins (ΔGsix+PTX) is inactivated (**i**). Shown are representative FRET traces with the indicated number of total cells per condition (**b**, **f**, **g**) and bar diagrams are averages from three independent experiments. Data are mean ± s.e.m. (**a**, **c**, **d**, **h**,** i**) or + s.d. (**b**, **e**, **f**,** g**) of three (**a**–**g**, **i**) and four (**h**) experiments (three technical replicates each). For statistical analysis, two-tailed unpaired *t*-test (**b**, **f**, **g**) and two-sample paired Wilcoxon test (**h**, **i**) was applied to paired points at different concentrations. *P < 0.05; **P < 0.01; not significant where no asterisk

**Fig. 2:**
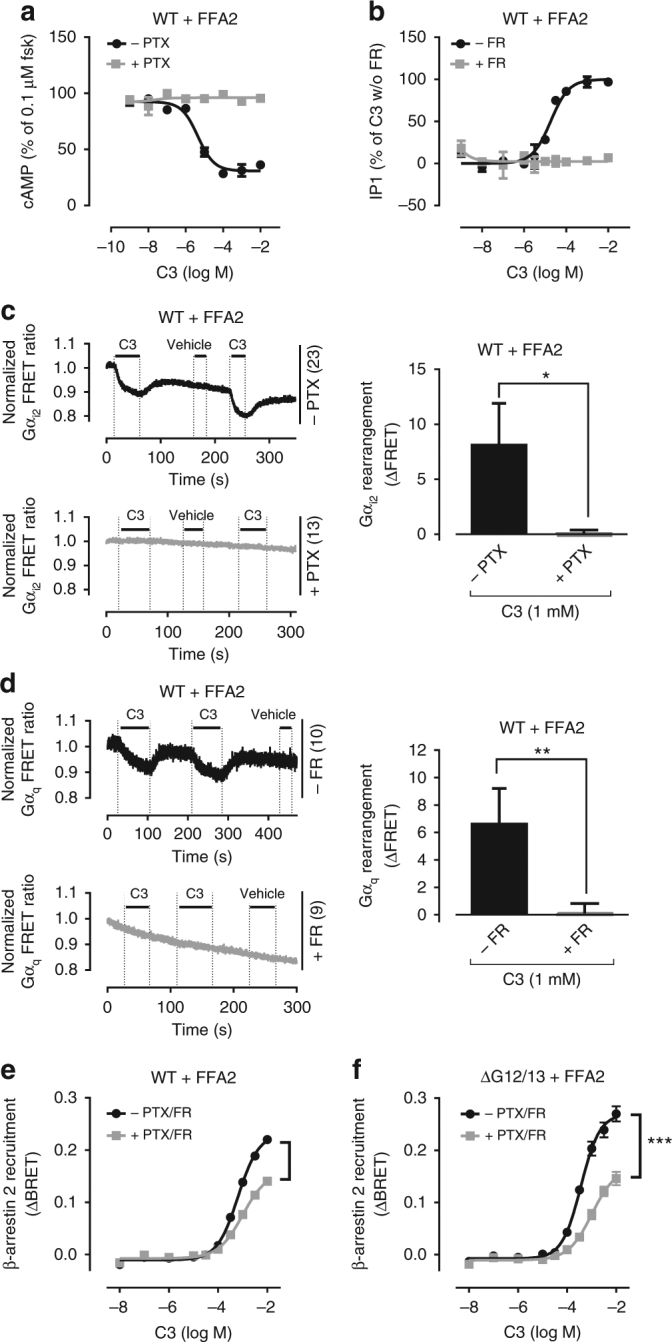
G protein activation and βarr2 recruitment in absence and presence of active G proteins. **a** Inhibition of forskolin-mediated cAMP accumulation by C3-activated FFA2 in FFA2-HEK293 cells, in the presence or absence of PTX. **b** IP1 accumulation of C3-stimulated FFA2, in the presence or absence of FR. **c**, **d** FRET measurements of Gi (**c**) and Gq (**d**) activation-induced conformational changes in wild-type (WT) HEK293 cells expressing FFA2 after stimulation with C3 in absence and presence of G protein inhibitors. **e**, **f** C3-induced β-arrestin2 recruitment to FFA2 in wild-type (WT) (**e**) or ΔG12/13 HEK293 cells (**f**), in the absence and presence of PTX and FR. Shown are representative FRET traces with the indicated number of total cells per condition (**c**, **d**) and bar diagrams are averages from three independent experiments. Data are mean ± s.e.m. (**a**, **b**, **e**, **f**) or + s.d. (**c**, **d**) of three experiments each performed in triplicate. For statistical analysis, two-tailed unpaired *t*-test (**c**, **d**) and two-sample paired Wilcoxon test (**e**, **f**) was applied to paired points at different concentrations. *P < 0.05; **P < 0.01; ***P < 0.001; not significant where no asterisk

We assessed whether arrestin recruitment could be isolated from G protein signaling and activation-induced conformational changes within G protein heterotrimers for three class A GPCRs with different G protein-coupling profiles: D prostanoid receptor-2 (DP2, Gi-coupled)^33^, orphan GPR17 (Gi/q-coupled)^34^, and free fatty acid receptor-2 (FFA2, Gi/q/12-coupled)^35^). To examine interaction of receptors with canonically studied signaling partners in HEK293 cells, we utilized traditional second messenger assays, genetically encoded Förster resonance energy transfer (FRET)-based “activation biosensors” for Gi and Gq^36^, and bioluminescence resonance energy transfer-based (BRET) measurements of induced interaction between βarr2 and each receptor. As expected prostaglandin D_2_ (PGD_2_) lowered intracellular cAMP accumulation through DP2 in stable DP2-HEK293 transfectants, and this decrease was abolished when cells were pretreated with Gi/o inhibitor pertussis toxin (PTX) (Fig. 1a). PTX also blunted the decrease of FRET ratio obtained by PGD_2_-stimulated DP2 indicative of activation-induced conformational rearrangement within the Gi FRET sensor (Fig. 1b). In contrast, βarr2 recruitment by DP2 was preserved when Gi/o proteins were inactivated (Fig. 1c). G protein-independent arrestin recruitment was also found for Gi/q-linked orphan GPR17 upon stimulation with the surrogate agonist MDL29,951 (MDL). While cAMP depression and inositolphosphate (IP) accumulation was fully prevented by pretreatment of cells with either PTX or Gq/11 inhibitor FR900359 (FR), respectively (Fig. 1d, e), as were activation-induced decreases of FRET ratios for Gi and Gq conformational sensors, respectively (Fig. 1f, g), βarr2 recruitment was only slightly affected by concomitant Gi/q inhibition (Fig. 1h). Robust βarr2 recruitment was even preserved for GPR17 in the collective absence of all functional G proteins [ΔGsix (ΔGs ΔGolf ΔGq/11 ΔG12/13)+PTX] (Fig. 1i; see Supplementary Fig. 1 for validation of ΔGsix cells). Similarly, pharmacological Gi/o and Gq/11 inhibition fully blunted cAMP or IP1 production in response to propionic acid (C3) stimulation of FFA2 (Fig. 2a, b), and prevented the C3-mediated decrease of FRET ratios resulting from “activated” Gi and Gq biosensors (Fig. 2c, d). However, combined inhibitor treatment did not suffice to eliminate βarr2 recruitment in the presence (Fig. 2e) or genetic absence of Gα_12/13_ (ΔG12/13; Fig. 2f). Thus, βarr2 recruitment could indeed be decoupled from G protein signaling, a common pattern observed for all three sample receptors. Hereafter, we refer to this phenomenon as “βarr recruitment at zero functional G”.

### Label-free biosensing detects G protein but not arrestin signaling

**Fig. 3:**
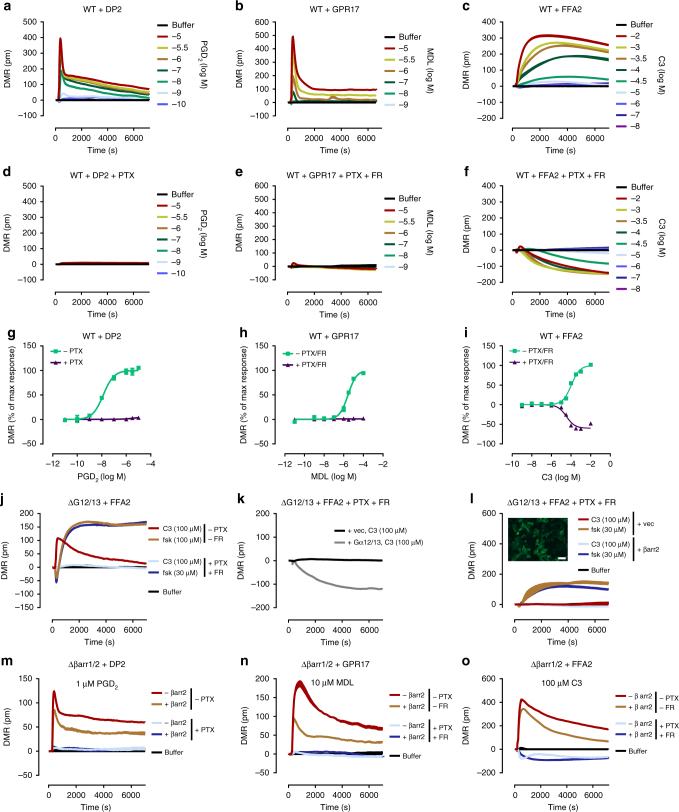
DMR biosensing of G protein- and arrestin-mediated cellular responses. DMR recordings in absence (**a**–**c**) and presence of G protein inhibitors (**d**–**f**) of agonist-mediated DP2 (**a**, **d**), GPR17 (**b**, **e**) and FFA2 (**c**, **f**) in wild-type (WT) HEK293 cells. Concentration-response-curves of DMR peak values in absence and presence of G protein inhibitors of DP2 (**g**), GPR17 (**h**), and FFA2 (**i**) response in wild-type HEK293 cells. **j** C3-mediated and forskolin-mediated DMR response of FFA2 in ΔG12/13 cells in the absence and presence of PTX/FR. **k** C3-induced DMR response of FFA2 in vector- or Gα_12/13_-transfected ΔG12/13 cells. **l** C3- or forskolin-mediated DMR response of FFA2 in vector- or β-arrestin2-transfected ΔG12/13 cells in the presence of PTX/FR. Inset shows βarr2-GFP transfected ΔG12/13 cells, scale bar 50 µm. **m**–**o** Agonist-induced DMR response of DP2 (**m**), GPR17 (**n**), or FFA2 (**o**) in vector- or β-arrestin2-transfected Δβarr1/2 cells in absence and presence of G protein inhibitors. **a**–**f**, **j**–**o** Shown are representative traces (mean + s.e.m.) of three independent experiments, each performed in triplicate. **g**–**i** Data are mean ± s.e.m. of three independent experiments (three technical replicates)

β-arrestin recruitment to a receptor in the absence of active G proteins is a captivating concept previously linked to numerous fundamental cellular processes such as cytoskeletal reorganization and chemotaxis among others^12,37,38^. Therefore, we reasoned that technology platforms competent to portray stimulus-dependent cell shape changes in response to diverse cellular signaling pathways should be ideal to visualize the cellular consequences that originate from “βarr recruitment at zero functional G”. Herein, we performed real-time live cell sensing of morphological changes with an optical biosensor based on detection of dynamic mass redistribution (DMR)^30^. One remarkable feature of DMR biosensing is the capacity to illustrate the activation of all four major G protein pathways (Gi/q/s/12)^31,32^, although whether DMR also visualizes G protein-independent, arrestin-dependent processes is elusive at present. We found robust and concentration-dependent DMR signatures for ligand-activated DP2 (Fig. 3a), GPR17 (Fig. 3b), and FFA2 (Fig. 3c) that were abolished when Gi activation of DP2 (Fig. 3d) or Gi/q activation of GPR17 (Fig. 3e) were ablated with PTX and/or FR, respectively, in agreement with their established signaling profiles. In contrast, ascending DMR signals elicited by C3-stimulated FFA2 were decaying slowly upon pretreatment with PTX and FR (Fig. 3f), indicative of a Gi/q-independent signaling event for this receptor (see Fig. 3g–i for concentration-effect curves describing the DMR responses for all receptors and Supplementary Fig. 2 for validation of inhibitor treatment). Because FFA2 is also coupled to G12/13 we next used HEK293 cells null for either Gα_12_ and Gα_13_ (ΔG12/13) or null for βarr1 and βarr2 (Δβarr1/2) to dissociate these signaling options. We found that negative DMR traces of C3-stimulated FFA2 were absent in ΔG12/13 HEK cells (Fig. 3j), were rescued by exogenous transfection of Gα_12_ and Gα_13_ (Fig. 3k), but did not re-emerge upon enrichment of β-arrestin2 (Fig. 3l). Thus, morphological cell shape changes were entirely driven by heterotrimeric G proteins because conditions that eliminated G protein signaling were required and sufficient to abolish DMR. In agreement, HEK293 cells depleted of both βarr1 and βarr2 (Δβarr1/2) retained robust DMR responses that again displayed the expected G protein inhibitor sensitivity profiles for each of the three receptors (Fig. 3m–o, compare red with light blue traces). Moreover, re-expression in Δβarr1/2 cells of exogenous βarr2 dampened ascending real-time DMR signals of all receptors consistent with a central role of arrestins in receptor desensitization (Fig. 3m–o, compare red with beige traces). From these data, we concluded that G proteins but not arrestins were genuine initiators of cell morphology changes because conditions that abolished DMR traces did not eliminate βarr recruitment for all tested receptors (compare Figs. 3d, e, j with 1c, h and 2f). Nevertheless, arrestin action downstream of G protein signaling was clearly evident and reflected in decreased DMR amplitudes, consistent with the central role of arrestins as desensitizers of G protein-mediated signal transduction. Apparently, holistic DMR detection is “blind” to βarr signaling at “zero functional G” despite its validated capacity to monitor fundamental cellular processes such as adhesion, proliferation, migration, and signal transduction^31,32,39–41^.

### G proteins dictate the phosphorylation of ERK1/2

**Fig. 4:**
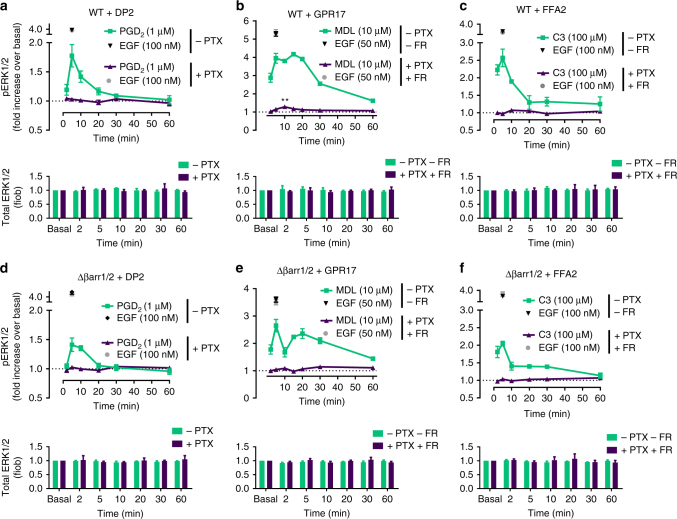
G protein vs. βarr1/2 dependence of ligand-stimulated ERK1/2 phosphorylation. **a**–**c** Kinetic pERK1/2 and total ERK1/2 profile of PGD_2_-stimulated DP2 (**a**), MDL-activated GPR17 (**b**), and C3-stimulated FFA2 (**c**) in the absence and presence of PTX and FR in wild-type (WT) HEK293 cells. **d**–**f** Temporal pattern of pERK1/2 and total ERK1/2 for PGD_2_-stimulated DP2 (**d**), MDL-stimulated GPR17 (**e**), and C3-stimulated FFA2 (**f**) in Δβarr1/2 cells in the absence and presence of PTX and FR. Data are mean +/± s.e.m. of three (**b**, **c**, **f**) and four (**a**, **d**, **e**) independent experiments (3 technical replicates each). For statistical analysis, one-sample *t*-test (**b**) was performed. **P < 0.01

One of the earliest discovered and most studied arrestin-dependent signaling pathways is phosphorylation of the ERK1/2 cascade^5,11,42–44^. Consequently, we asked whether G protein-independent arrestin recruitment translated to ERK1/2 phosphorylation downstream of our sample receptors. Time course experiments revealed rapid increases of ERK1/2 phosphorylation that decreased to baseline or sustained low amounts with distinct kinetic profiles for individual receptors (Fig. 4a–c). Pharmacological elimination of Gi or Gi/q signaling blunted ERK1/2 phosphorylation mediated by PGD_2_-stimulated-DP2 and C3-activated FFA2, respectively, and largely eliminated but did not abolish the early ERK1/2 peak induced by MDL-activated GPR17 (Fig. 4b). The early ERK1/2 peak was lost, however, when cells were depleted of all functional G proteins using combined genetic ablation (ΔGsix) and pharmacological inhibition (PTX) (Supplementary Fig. 3). Conversely, ERK1/2 was phosphorylated downstream of all receptors in cells lacking β-arrestin1/2 (Fig. 4d–f) (see refs. ^24,29^ for additional validation and characterization of genome-edited Δβarr1/2 cells). As anticipated, pharmacological silencing of G protein activity in Δβarr1/2 cells was required and sufficient to abolish ERK1/2 activation at all time points for our receptor panel (Fig. 4d–f). Note that treatment of cells with the G protein inhibitors PTX and FR did not globally perturb MAPK activation because epidermal growth factor (EGF) responses were essentially unaltered under all treatment regimens (Fig. 4, Supplementary Fig. 3). We noted a modest and at times significant attenuation of ERK1/2 phosphorylation for DP2 and GPR17 in Δβarr1/2 cells compared with the effect in wild-type HEK293 cells (Supplementary Fig. 4a–c), likely indicative of β-arrestin's scaffold function for MAP kinase signaling downstream but not independent of G proteins. In line with this notion are somewhat higher median effective concentrations (EC_50_) of DP2 and GPR17 agonists for ERK1/2 phosphorylation in Δβarr1/2 cells (Supplementary Fig. 4d–f). Surface abundance for all three receptors is comparable between wild-type and Δβarr1/2 HEK cell lines (Supplementary Fig. 4g–i), indicating that quantitative differences in pERK signaling are not related to difference in receptor surface expression levels. From these data we concluded that both early and late phase ERK1/2 activity was driven by G proteins but not arrestins for all three receptors. Apparently, arrestin recruitment at “zero functional G” is not linked to ERK1/2 signaling in this cellular background for the studied receptors.

### Prototype GPCRs for βarr-mediated signaling drive pERK via G proteins

**Fig. 5:**
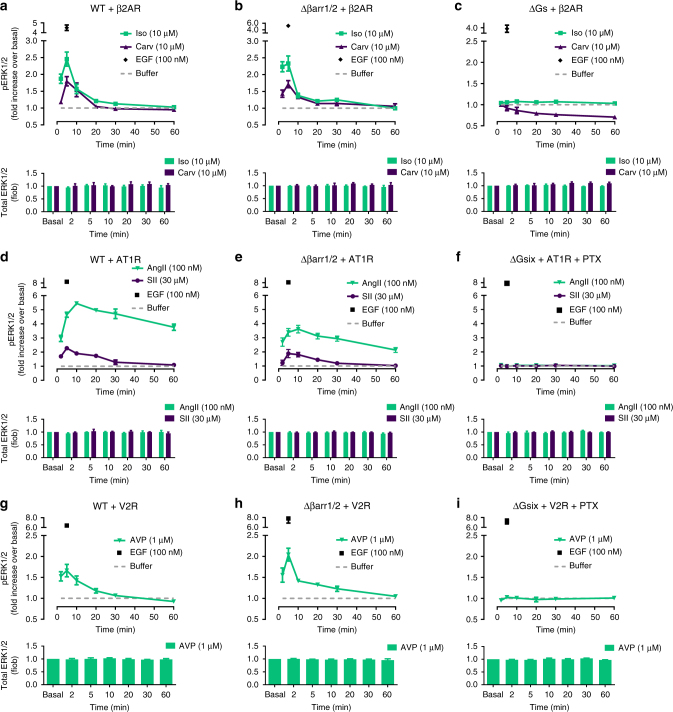
pERK1/2 profiles of ligand-activated β2AR, AT1R, and V2R in WT and Δβarr1/2 cells. **a**–**c** Temporal pattern of ERK1/2 phosphorylation and total ERK1/2 by β2AR in wild-type (**a**), Δβarr1/2 (**b**), and ΔGs (**c**) HEK293 cells treated with the indicated ligands. **d**–**f** Temporal pattern of ERK1/2 phosphorylation and total ERK1/2 by ligand-activated AT1R in wild-type (**d**), Δβarr1/2 (**e**), and ΔGsix cells+PTX (**f**). **g**–**i** Kinetic pERK1/2 and total ERK1/2 profile of V2R in wild-type (**g**), Δβarr1/2 (**h**), and ΔGsix cells+PTX (**i**). Data are mean +/± s.e.m. of three (**g**, **h**, **i**), four (**a**–**d**, **f**) and five (**e**) experiments each performed in triplicate

**Fig. 6:**
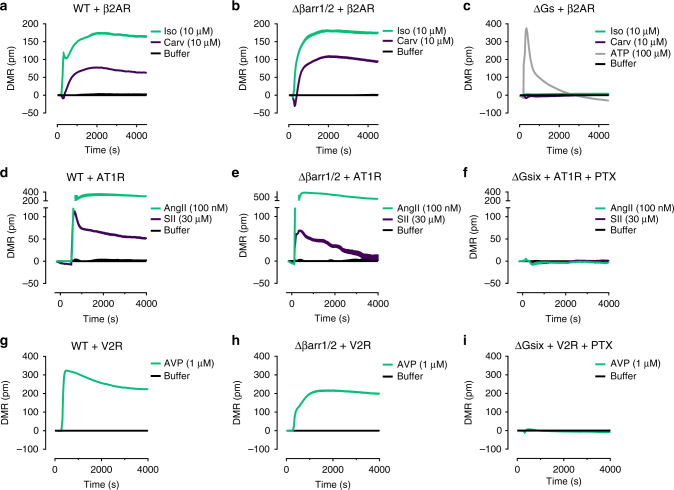
DMR profiles of ligand-activated β2AR, AT1R, and V2R in WT and Δβarr1/2 HEK cells. **a**–**c** DMR recordings of β2AR in wild-type (**a**), Δβarr1/2 (**b**), and ΔGs (**c**) cells treated with Isoproterenol (Iso) or Carvedilol (Carv). **d**–**f** DMR recordings of AT1R wild-type (**d**), Δβarr1/2 (**e**) and ΔGsix cells+PTX (**f**) treated with AngII or SII. **g**–**i** DMR phenotyping of V2R in wild-type (**g**), Δβarr1/2 (**h**), and ΔGsix cells+PTX (**i**). DMR traces shown are representative (mean + s.e.m.) for one out of three independent experiments, each performed in triplicate

To prevent sampling bias and because traditional pathway-specific and innovative label-free cellular readouts revealed G protein-dependency of cellular signaling for all investigated receptors, we broadened our analysis to three prototype class A GPCRs: the β2-adrenergic (β2AR), the angiotensin II type 1 A (AT1R), and the V2 vasopressin receptor (V2R), all of which were reported to also signal in a β-arrestin-dependent, G protein-independent manner^5,7,11,37,45^. β2AR is classified as a transient arrestin-binding class A, AT1R, and V2R as stable arrestin-binding class B GPCRs^46^. We found similar ERK1/2 phosphorylation profiles when Gs-coupled β2AR was stimulated with saturating concentrations of the synthetic full agonist isoproterenol (Iso), and carvedilol (Carv), which is classified by some as both nonselective β-blocker but partial agonist for β-arrestin-mediated, G protein-independent ERK1/2 signaling^8^ (Fig. 5a). ERK1/2 phosphorylation in response to both stimuli was preserved in cells lacking arrestins (Δβarr1/2, Fig. 5b and Supplementary Fig. 5a, b) but not in cells lacking Gs proteins (ΔGs, Fig. 5c). Comparable β2AR abundance was confirmed across all cell lines using surface ELISA (Supplementary Fig. 6). These results suggested that Gs proteins but not arrestins are key components for initiation of β2AR-mediated MAPK signaling. Similarly, ERK1/2 was phosphorylated when the AT1R was stimulated with the natural agonist angiotensin II (AngII) or the synthetic analog [Sar^1^, Ile^4^, Ile^8^]AngII (SII) (Fig. 5d), reported to function as “completely biased agonist” for the arrestin pathway in HEK293 cells^11,44^. Again, this effect was not driven by arrestins (Fig. 5e and Supplementary Fig. 7a, b) but entirely depended on G proteins (Fig. 5f). Similar findings were obtained for V2R, another GPCR described by some as prototypal for arrestin-dependent, G protein-independent MAP kinase signaling upon stimulation with its endogenous ligand arginine vasopressin (AVP; Fig. 5g–i and Supplementary Fig. 7c)^45^. Although our data do not support G protein-independent, arrestin-dependent ERK1/2 phosphorylation by β2AR as postulated previously, they do attest to the role of arrestins as ERK signaling scaffolds. In fact, a gradual increase of βarr2-GFP abundance using gene dosing in Δβarr1/2 cells revealed unaltered kinetic profiles for ERK1/2 phosphorylation with low βarr2 amounts, enhanced signal amplitude and duration with increased βarr2 amounts but attenuation of ERK signaling at high βarr2 abundance (Supplementary Fig. 8). Bell-shaped dependence of signaling on scaffold concentration is a hallmark feature of signaling scaffolds, i.e., proteins that bring signaling components together but do not activate them^47–49^. We, therefore, appreciate that arrestin scaffolding imposes constraints on kinase activity that dictate ERK signal amplitude and duration, but propose that this behavior implies arrestin action downstream but not independent of G proteins. G protein but not arrestin-driven signaling was also found in DMR recordings for the three receptors, congruent with the findings obtained for our previous panel of family A GPCRs. Whole-cell DMR recordings revealed global cell responses for isoproterenol and carvedilol in wild-type HEK293 cells (Fig. 6a). In Δβarr1/2 cells integrated DMR responses remained detectable for both ligands (Fig. 6b). Conversely, signaling capacity was lost for both isoproterenol and carvedilol when Gs proteins were deleted (ΔGs) (Fig. 6c). Signaling patterns similar to those observed with the β-agonists were also found for the AT1R upon challenge with AngII and SII (Fig. 6d–f), and for AVP-activated V2R (Fig. 6g–i). Note the altered DMR response of AVP in Δβarr1/2 cells compared with that in wild-type HEK293 cells, consistent with the role of arrestins to promote and extend cAMP signaling of this receptor^16^. Thus, DMR biosensing and ERK1/2 phosphorylation did not reveal βarr signaling at “zero functional G”, a cellular event neither driving morphology changes nor ERK1/2 signaling by our sample receptors.

### DREADDs reveal G proteins as drivers for pERK1/2 and holistic DMR

**Fig. 7:**
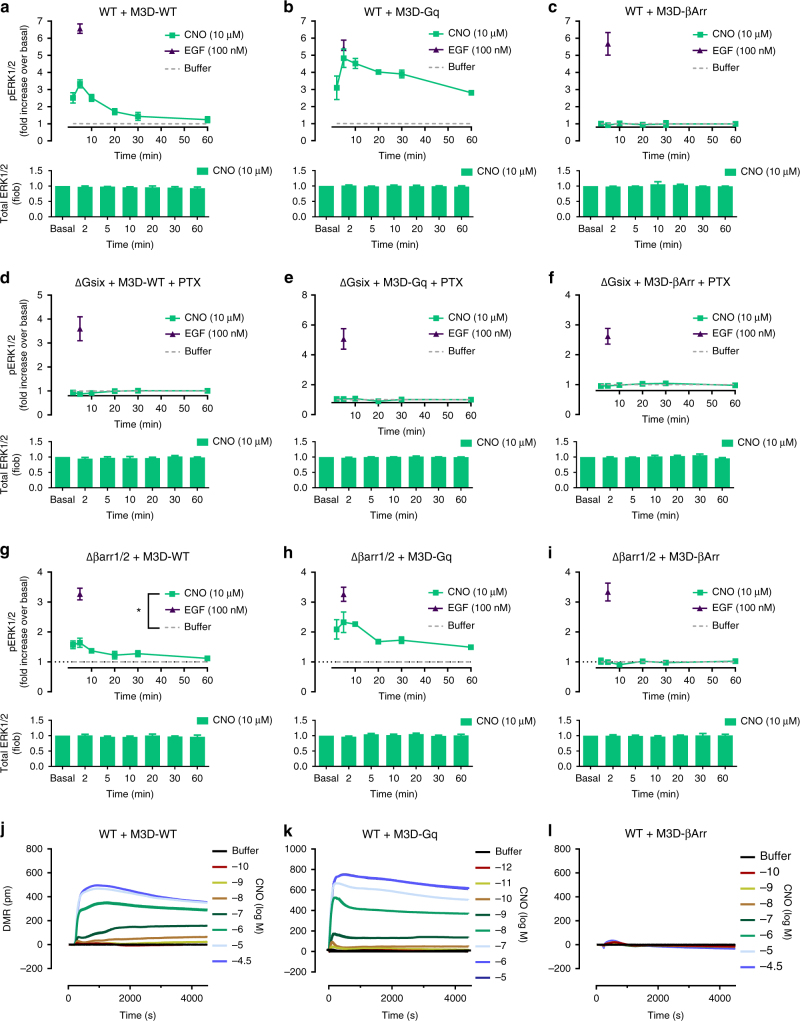
Designer receptor-mediated ERK1/2 activation and DMR response. **a**–**c** Clozapine-N-oxide (CNO)- and epidermal growth factor (EGF)-mediated ERK1/2 phosphorylation and total ERK1/2 in wild-type HEK293 cells (WT) expressing M3D-WT (**a**), M3D-Gq (**b**) or M3D-βArr (**c**). **d**–**f** CNO- and EGF-mediated ERK1/2 phosphorylation and total ERK1/2 in G protein-deficient (ΔGsix+PTX) HEK293 cells by M3D-WT (**d**), M3D-Gq (**e**) or M3D-β-arr (**f**). **g**–**i** CNO-mediated and EGF-mediated ERK1/2 phosphorylation and total ERK1/2 in β-arrestin-deficient (Δβarr1/2) cells by M3D-WT (**g**), M3D-Gq (**h**) or M3D-βArr (**i**). **j**–**l** Real-time DMR traces in CNO-stimulated cells expressing M3D-WT (**j**), M3D-Gq (**k**) or M3D-βArr (**l**). **a**–**i** Data are mean +/± s.e.m. of three independent experiments (three technical replicates). **j**–**l** Shown are representative traces (mean + s.e.m.) of three independent experiments, each measured in triplicates. For statistical analysis, two-sample paired Wilcoxon test (**g**) was applied to paired points at different times. *P < 0.05

We next used designer receptors that are exclusively activated by designer drugs (DREADDs) to attempt dissociation of G protein from arrestin signaling in our CRISPR cell lines. Of this family of evolved muscarinic M3 receptors we used three variants: an “unbiased” (M3D-WT), a “Gq-biased” (M3D-Gq), and an “arrestin-biased” (M3D-βArr) variant (designated as such according to their signaling preferences in HEK293 and COS7 cells, respectively^50–52^). All three receptors are poorly responsive to acetylcholine but can be efficiently activated by clozapine N-oxide (CNO), a clozapine analog essentially inert at wild-type M3 receptors^50^. As anticipated, CNO led to robust ERK1/2 phosphorylation over time in HEK293 cells expressing the G protein-coupled DREADDs, M3D-WT and M3D-Gq (Fig. 7a, b). In contrast, M3D-βArr, the G protein-uncoupled but arrestin-preferring variant, was completely inactive despite robust surface abundance and arrestin recruitment (Fig. 7c, Supplementary Fig. 9a, b). Consistent with these findings, collective depletion of all active Gα proteins (ΔGsix+PTX) abolished MAPK signaling for all three DREADDs suggesting that G protein-independent mechanisms did not contribute to ERK1/2 activation in this system (Fig. 7d–f). Note that cells without active Gα responded to EGF with robust ERK1/2 phosphorylation, albeit reduced in relation to wild-type (Fig. 7d–f), expressed all DREADDs at comparable amounts (Supplementary Fig. 10a), and showed β-arrestin2 translocation upon CNO treatment for M3D-WT and M3D-βArr (Supplementary Fig. 10b). In contrast, cells lacking βarr1/2 maintained ERK1/2 phosphorylation only for the G protein-coupled DREADDs (Fig. 7g–i). Although sustained ERK signaling was apparent in Δβarr1/2 cells for M3D-Gq as compared with M3D-wt, this likely relates to enhanced surface abundance of this receptor variant (Fig. 7g, h, and Supplementary Fig. 11). DMR assays endorsed the essential role of G proteins as cell signaling elements because only G protein-coupled DREADDs produced global cellular activity in response to CNO (Fig. 7j–l, Supplementary Fig. 12). Thus, using DREADDs to parse contribution of G proteins vs. arrestins in ERK1/2 and holistic DMR assays unveiled a common theme: βarr recruitment at “zero functional G” was a salient feature of M3D-βarr and an inherent feature of M3D-wt and various different class A GPCRs, but was neither required nor sufficient to drive ERK signaling or higher-order cellular processes such as morphology changes in its own right.

### G protein-driven pERK is no epiphenomenon of a single Δβarr1/2 clone

**Fig. 8:**
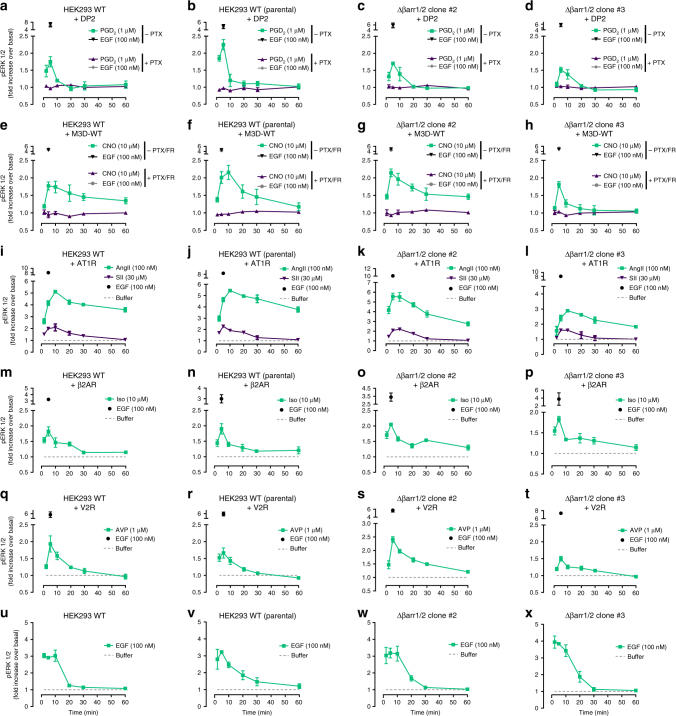
Ligand-stimulated pERK1/2 profiles in wild-type HEK293 and Δβarr1/2 cells. Kinetic pERK1/2 profiles of agonist-stimulated DP2 (**a**–**d**), M3D-WT (**e**–**h**), AT1R (**i**–**l**), β2AR (**m**–**p**), V2R (**q**–**t**), and GPCR-independent MAPK pathway activation by EGF (**u**–**x**) in wild-type (WT) (**a**, **e**, **i**, **m**,** q**, **u**), wild-type (WT) (parental) (**b**, **f**, **j**, **n**, **r**, **v**), Δβarr1/2 clone #2 (**c**, **g**, **k**, **o**, **s**, **w**), or Δβarr1/2 clone #3 (**d**, **h**, **l**, **p**, **t**, **x**). Data are mean ± s.e.m. of three independent experiments (three technical replicates)

It is known that transformed cells can drift upon multiple passages, growth conditions and removal of genes and even modify their response behaviors and means to engage specific signaling pathways^53,54^. We therefore broadened our analysis by two independent CRISPR/Cas9 Δβarr1/2 clones (hereafter clone#2 and clone#3, all previous data generated on clone#1^24^) and one additional wild-type HEK293 clone, distinct from the parental HEK293 line used for generation of the CRISPR/Cas9 genome-edited cells. We initially confirmed absence of βarr1 and βarr2 in the three Δβarr1/2 HEK293 lines by immunoblot using polyclonal antibodies recognizing the C-termini of both proteins (Supplementary Fig. 13). We then investigated the contributions of G proteins and arrestins to ERK1/2 MAPK signaling using the same stimuli and receptors employed before. PGD_2_-stimulated DP2 promoted ERK1/2 phosphorylation that peaked at early time points to then rapidly decay to basal amounts in wild-type and Δβarr1/2 clone #2 and #3 (Fig. 8a–d). Again, ERK1/2 phosphorylation was driven by G proteins across all cell lines because PTX pretreatment but not genetic ablation of arrestins abolished the pERK1/2 response (Fig. 8a–d). CNO-activated M3D-WT also required G proteins for ERK1/2 signaling (Fig. 8e–h) although we noted a somewhat distinct kinetic pattern of phosphorylated ERK1/2 in Δβarr1/2 clone #3. Nevertheless, kinetics alone are insufficient to distinguish between G protein- and arrestin-dependent pERK1/2^10,24,55,56^. Similar findings were obtained for the three prototype receptors AT1R (Fig. 8i–l), β2AR (Fig. 8m–p) and V2R (Fig. 8q–t): agonist-mediated ERK1/2 phosphorylation was retained when cellular arrestins were deleted. EGF-activated MAPK signaling was similar across all clones (Fig. 8u–x) although we noted considerable variation upon expression of exogenous GPCRs in individual clones. Global cell responses using a panel of receptor-dependent and -independent stimuli did not indicate functional abnormality of any of the arrestin null lines (Supplementary Fig. 14). Moreover, absence of receptor-mediated internalization in Δβarr1/2 clones #1-#3 for both AVP-stimulated V2R and isoproterenol-activated β2AR that was rescued upon re-introduction of arrestins further validated the genome-edited cells (Supplementary Fig. 15). From these data, we concluded that signaling pathway usage for GPCR-mediated ERK1/2 phosphorylation is uniform for all receptors studied in this cellular background: G proteins acted as drivers and arrestins as scaffolds downstream of active G proteins that may determine signal intensity and duration of phosphorylated ERK1/2. Thus, G protein-dependence of ERK1/2 phosphorylation did not result from clonal heterogeneity of wild-type and genome-edited HEK293 cells.

### βarr recruitment at “zero functional G” regulates GPCR surface levels

**Fig. 9:**
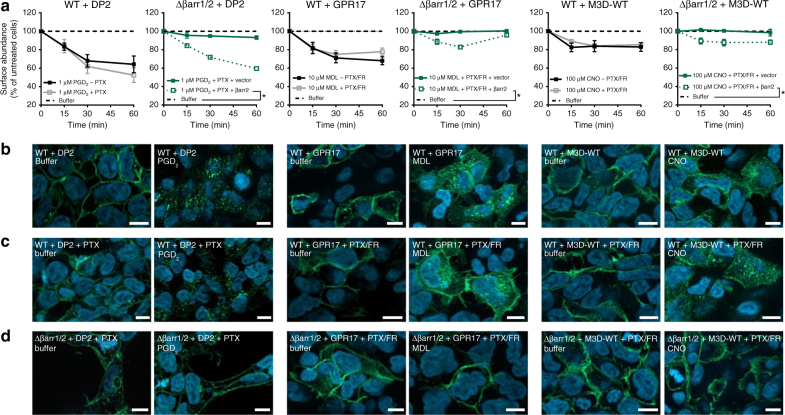
Ligand-mediated GPCR internalization in the absence of G proteins and/or arrestins. **a** Kinetic surface ELISA of buffer-treated or agonist-treated HEK293 cells expressing DP2 (FLAG-tagged), GPR17 (HA-tagged), and M3D (HA-tagged) receptors in the presence and absence of PTX and FR in wild-type (WT) and Δβarr1/2 cells. **b**–**d** Structured-illumination micrographs of buffer or agonist-treated receptors in wild-type HEK293 (**b**, **c**) or Δβarr1/2 cells (**d**) in the absence (**b**) or presence (**c**, **d**) of PTX and FR. Data are mean ± s.e.m. of three independent experiments, each performed in triplicate (**a**). Merged pictures of DAPI-stained nuclei (blue) and receptor-targeted Cy2-tagged antibody (green), scale bar is 10 µm; representative of three independent experiments (**b**–**d**). For statistical analysis, two-sample paired Wilcoxon test (**a**) was applied to paired points at different times. *P < 0.05

Since we were unable to detect a functional correlate for arrestin recruitment in the absence of active G proteins, we next asked whether this biological process might be linked to a non-signaling function, such as an alternative means to regulate surface abundance of GPCRs. To this end, we investigated internalization of ligand-stimulated receptors when G proteins were pharmacologically inhibited with PTX and/or FR or genetically ablated. We performed surface ELISA of either HA-tagged (GPR17, M3D-wt) or FLAG-tagged (DP2) receptors, live cell imaging of YFP-tagged (FFA2) and immunostaining of fixed cells (GPR17, M3D-WT, DP2). As anticipated, all receptors internalized over time in agonist-stimulated wild-type HEK293 cells as assessed by ELISA (Fig. 9a, black solid lines) or immunofluorescence microscopy (Fig. 9b and Supplementary Fig. 16a). Ligand-induced internalization was not ablated when interacting G proteins were specifically inhibited and/or eliminated for each receptor as assessed by either ELISA (Fig. 9a, gray solid lines) or structured-illumination imaging (Fig. 9c, and Supplementary Fig. 16b). Concurrent deletion and/or functional inactivation of both arrestins and G proteins precluded receptor internalization (Fig. 9d), an effect that was reversed for all three receptors when β-arrestin2 was re-introduced into the βarr1/2 null cells (Fig. 9a, compare green dotted with solid lines). These data indicated that arrestins were essential mediators of internalization when G protein activation was inhibited, thereby highlighting and expanding their role from desensitizers of G protein-dependent signaling to regulators of GPCR surface abundance under conditions when receptors are active but G proteins are not.

## Discussion

What happens when G protein-coupled receptors are active but their associated G proteins are all inactive or collectively eliminated? What does signaling at “zero functional G” look like on a cellular level and can we visualize arrestin-dependent, G protein-independent signaling using a label-free, real-time integrated readout for global cellular activity? Albeit intriguing, answers remained elusive in large part ascribed to the lack of tools to accomplish collective absence or functional inactivity of Gα isoforms from all four major G protein families, and arrestins, respectively. Arrestins have been viewed by some as core components of G protein-independent signaling to the ERK1/2 MAP kinase cascade^12,42,57^. Although we expected phosphorylation of ERK1/2 MAP kinases to persist in the absence of active G proteins, we found instead sustained recruitment of arrestin for all receptors irrespective of their G protein-coupling profiles, but failure to activate ERK1/2 and induce morphology changes, which are common signaling attributes of cell surface GPCRs^7,42,44,45,58^. β-arrestin independence for initiation of ERK phosphorylation has recently been demonstrated for the β2AR using various complementary genetic approaches in the HEK293 cell background^29^. We here significantly expand this concept by showing that βarrs are non-essential for driving ERK MAPK signaling for a broad panel of family A GPCRs. This even applies to the three prototypical receptors β2AR, AT1R, and V2R, all of which have been previously reported to activate MAP kinases also through β-arrestins^5,7,11,45^. These data change our understanding of how GPCRs function and in particular how they activate ERK1/2.

Intriguing questions arise from these observations: Why do our conclusions contrast with the large body of evidence obtained in pioneering studies more than a decade ago, despite the same cellular background? Why does label-free phenotypic DMR biosensing not visualize βarr signaling at “zero functional G” or more generally any functional correlate of G protein-independent signaling on a whole-cell level? Why has chemotaxis in HEK293 cells with stable expression of the AngII receptor AT1R been previously defined as βarr2-dependent and apparently G protein-independent^37^, yet AT1R-expressing cells fail to induce cell morphology changes when G proteins are inactive (this study)?

What appears as surprising discordance at first glance may be explained by noting some key differences between the current and previous studies. First, it has not been technically possible to completely eliminate arrestins or G proteins, thus posing challenges to data interpretation^7,8,10,11,37,43,44^. In fact, numerous previous studies reporting G protein-independent, arrestin-dependent ERK1/2 activation were based on incomplete suppression of arrestin abundance using siRNA or shRNA^7,8,11,43,44^. Remarkably, even these have not provided a consensus view: reduced^8^, unaltered^21^, or enhanced^29^ pERK1/2 was observed for ligand-stimulated β2AR in different laboratories. Whether such experimental disparities are attributable to knockdown of non-targeted proteins, potentially undermining data interpretation, or genetic drifts that are known to occur in cells upon multiple passages, and that may impact their response behaviors and means to engage specific signaling pathways^53,54^ is elusive at present. The few studies using mouse embryonic fibroblasts that are null for G proteins and/or arrestins have also led to conflicting results concerning mechanisms of ERK1/2 activation^7,28,59–61^. The current study was conducted with CRISPR/Cas9 genome-edited HEK293 cell lines in which signaling adaptors were inactivated by either genetic ablation (arrestins) or combined genetic and pharmacological inhibition (ΔGsix (ΔGs ΔGolf ΔGq/11 ΔG12/13)+PTX) and should therefore be less ambiguous. Although no fully G protein-ablated cell line has been created, “ΔGsix+PTX” conditions (aka “zero functional G”) allowed for the first time interrogation of signaling events that occur in the collective absence of functional G proteins from the four major families. Together with arrestin null cells, we expect ΔGsix cells to be of great value for classification of signaling pathways to be genuinely initiated by either arrestins or G proteins.

Second, elimination of G protein signaling has not been previously possible except for members of the Gi/o family that were reliably inactivated by PTX. This is particularly relevant when interpreting functional outcomes of the AT1R which is coupled to Gi/o, Gq/11, and G12/13^56^. Resilience of a functional AT1R signal in the presence of PTX and siRNA for Gα_q/11_ therefore does not necessarily indicate G protein-independent signaling because the G12 family may have contributed to the overall signaling outcome, and residual amounts of Gα_q/11_ may have been sufficient to produce robust signals particularly for amplified measures of receptor activation such as ERK1/2 phosphorylation or chemotaxis^37^. To date, specific and quantitative inhibition of Gq/11 signaling can be achieved with FR900359 or YM254890, two depsipeptides from natural sources that are commonly applied to inhibit these proteins^24,26,62^ and that ideally complement the genome-edited cells.

Third, one of the first and most widely used “arrestin-biased” ligands, the AngII peptide analog SII, was initially classified as arrestin-biased agonist unable to activate G proteins but is now recognized also as low efficacy partial agonist for G protein signaling^56,63^. In fact, our data agreed well with the partial agonist nature for G protein activation of SII^56,63^ and also with the capacity of AngII to promote ERK1/2 phosphorylation in mouse embryonic fibroblasts genetically deficient for βarr1/2^56^. It is unfortunate that SII is still referred to as “completely biased” toward the arrestin arm of signaling^64^ despite its proven capacity to also activate the less preferred G protein arm^56,63^. A thorough mechanistic understanding of how AT1R activates ERK would require consideration of the full spectrum of biological activities SII exerts within a given cell type. Regardless, CRISPR/Cas9 lines null for G proteins or arrestins should prove extremely useful to assess the relative contributions of each of the different pathways to the ultimate biological variable that is being measured for SII or any other “biased ligand”.

One caveat deserves particular mention here: all results have been generated in HEK293 cells and we are aware that translation to other cell types, primary cells or even whole animals must be performed with caution. Conceivably, cell-dependent, tissue-dependent, and context-dependent differences such as relative abundance of β-arrestin isoforms or G protein-coupled receptor kinases may add further complexities to shape the final signaling output^65,66^. Notwithstanding, arrestin-dependent, G protein-independent ERK activation has been proposed in this very cellular context selected by us for genetic manipulations.

Although we failed to establish a mechanistic link between G protein-independent βarr recruitment and ERK signaling or more generally, occurrence of phenotypic cell changes, we propose a biological purpose for βarr recruitment at zero functional G: control of surface abundance of GPCRs. Future studies will be needed to address whether “βarr recruitment at zero functional G” prevents cells from overstimulation and thereby serves a non-signaling function or rather enables new signaling from inside the cell. Along the same lines, truly arrestin-biased ligands that are devoid of G protein signaling may simply exert their biological effects through forced endocytosis and thereby act as functional antagonists, an outcome just opposite to the signaling action that was originally intended. Either way, CRISPR/Cas9 genome-edited cells with quantitative absence of functional Gα proteins or arrestins along with highly selective inhibitors of Gα protein function have been^24,67^ and will be key to answer such questions.

While we eagerly await future studies that determine how the lack of G proteins vs. arrestins affects the many aspects of GPCR biology, we here highlight the urgent need to reconsider our current perception of some basic principles in GPCR signal transduction. GPCRs are targets for 30% of prescription medicines; accordingly mechanisms through which they regulate cellular functions have major implications for development of novel drugs, including those that attempt to exploit independent G protein or arrestin pathways for therapeutic benefit.

## Methods

### Cell culture and chemicals

HEK293 (WT) were obtained from the American Type Culture Collection (ATCC), parental HEK293 (WT) and Flp-In T-REx293 were from ThermoFisher. HEK293 (WT), parental and CRISPR/Cas9 genome-edited HEK293 cells were cultured in DMEM (ThermoFisher) containing 10% fetal bovine serum (FBS, PAN biotech), 100 U ml^−1^ Penicillin, 100 mg ml^−1^ Streptomycin (ThermoFisher) at 37 °C and 5% CO_2_. Receptor expressing HEK293 cell lines were generated by stable transfection of receptor cDNA (GPR17, DP2, β2AR, AT1R, and M3-DREADD cloned into pcDNA3.1(+)) using Fugene HD (Promega) according to manufacturer's instructions and subsequently cultivated using growth medium containing 500 µg ml^−1^ G418 (Invivogen). FFA2 receptor was expressed in Flp-In T-REx293 cells upon induction of expression after treatment with 1 µg ml^−1^ doxycycline for at least 16 h. Transiently transfected cells were analyzed 24–48 h after transfection using Fugene HD (Promega). All cell lines were checked for and free of mycoplasma contamination. All chemicals were purchased from Sigma-Aldrich unless otherwise indicated. For G protein inhibition, cells were incubated with pertussis toxin (PTX) for at least 18 h at 150 ng ml^−1^ and FR for at least 1 h at 1 µM.

### Generation of CRISPR/Cas9 genome-edited HEK293 cells

Generation of genetically engineered HEK293 cells using CRISPR/Cas9 technology to knockout subunits of Gα_12_ and Gα_13_ (ΔG12/13), Gα_s_ and Gα_olf_ (ΔGs) is described elsewhere^22,25,29^. For generation of Δβarr1/2 cells, including clones #1, #2, and #3, see ref. ^29^; for generation and validation of Δβarr1/2 clone #1, see ref. ^24^. A combination of parallel Gα_q_, Gα_olf_, Gα_11_, Gα_s_, Gα_12_ and Gα_13_ knockout (ΔGs/olf/q/11/12/13=ΔGsix) and Gα_i/o_ inhibition with pertussis toxin (PTX, Biotrend) was used to determine total contribution of functional G proteins. ΔGsix were generated by simultaneously mutating the *GNAS* and the *GNAL* genes (encoding Gα_s_ and Gα_olf_, respectively) of previously established Gα_q/11/12/13_-KO HEK293 cells^68^, using a CRISPR/Cas9 system and sgRNA constructs targeting the *GNAS* and the *GNAL* genes^25^. The sgRNA-encoding sequence targeting the *GNAS* gene (5′-CTACAACATGGTCATCCGGG-3′) or the *GNAL* gene (5′-GTAATGTTTGCCGTCACCGG-3′) was inserted into the Bbs I site of the pSpCas9(BB)-2A-GFP vector (PX458; a gift from Feng Zhang, Broad Institute; Addgene plasmid # 48138). Briefly, the Gα_q/11/12/13_ KO HEK293 cells were seeded into 6-well plates and incubated for 24 h before transfection. A mixture of the *GNAS*-targeting vector (0.5 µg) and the *GNAL*-targeting vector (0.5 µg) was transfected into the cells using Lipofectamine 2000 (ThermoFisher) according to a manufacturer's protocol. Three days later, cells were detached and GFP-positive cells (~6% of cells) were isolated using a cell sorter (SH800, Sony). After growing clonal cell colonies with a limiting dilution method, clones were analyzed for mutations in the *GNAS* and the *GNAL* genes by PCR and restriction enzyme digestion. PCR conditions were as follows: initial denaturation cycle of 95 °C for 2 min, followed by 35 cycles of 95 °C for 15 s, 64 °C for 30 s, and 72 °C for 30 s. The resulting amplicon was verified by restriction enzyme digest. All cells were routinely tested for mycoplasma contamination by PCR detection method. HEK293 cells were used as they constitute one of the prototype cell systems, in which most basic research on β-arrestin-dependent and G protein-independent signaling has been studied in.

### SDS–PAGE and immunoblotting

Wild-type, ΔGq/11/12/13, ΔGsix, and Δβarr1/2 cells were lysed by adding lysis buffer (25 mM Tris, pH 7.4, 150 mM NaCl, 1 mM EDTA, 1% Triton X-100, 1% IGEPAL) complemented with protease inhibitor cocktail (Sigma) or SDS–PAGE sample buffer (62.5 mM Tris-HCl (pH 6.8), 50 mM dithiothreitol, 2% SDS, 10% Glycerol and 4 M urea) containing 1 mM EDTA and 1 mM phenylmethylsulfonyl fluoride. Lysates were agitated for 20 min at 4 °C and afterwards centrifuged at 13,200 rpm 4 °C for 10 min or were homogenized with a handy ultrasonic homogenizer (Microtech) and boiled at 95 °C for 5 min.

Equal amounts of protein were separated by 10–12.5% SDS-polyacrylamide gel electrophoresis. Subsequently, the proteins were transferred to nitrocellulose membrane (GE Healthcare). Membranes were blocked with Roti-Block (Carl Roth) (for antibodies against Gα_11/14/q,_ Gα_12_ and Gα_13_), 5% BSA in PBS buffer (0,1% Tween20) (for antibodies against Gα_i_ and Gα_s/olf_) or 5% skim milk in TBS buffer (0,05% Tween20) (for antibodies recognizing arrestin1 and arrestin2) for 30–60 min at room temperature and incubated overnight at 4 °C with the appropriate primary antibody in the following blocking solution Roti-Block for antibody against Gα_11/14/q_ (1:1000, cat. no. sc-365906 (G-7), Santa Cruz Biotechnology), Gα_12_ (1:1000, cat. no. sc-515445 (E-12), Santa Cruz Biotechnology) and Gα_13_ (1:1000, cat. no. sc-293424 (6F6-B5), Santa Cruz Biotechnology), 5% BSA in PBS buffer for antibodies specific for Gα_i_, (1:1000, cat. no. sc-262 (C-10), Santa Cruz Biotechnology) and Gα_s/olf_ (1:1000, cat. no. sc-55545 (A-5), Santa Cruz Biotechnology) or 1% BSA in TBS buffer for antibodies recognizing β-arrestin1 (1:1000_,_ cat. no. 12697 (D8O3J), Cell Signaling) and β-arrestin2 (1:1000_,_ cat. no. 3857 (C16D9), Cell Signaling).

To detect protein bands, membranes were washed three times and afterwards incubated for 30–60 min at room temperature with a horseradish peroxidase-conjugated secondary antibody, antibodies specific for rabbit IgG (1:10,000, cat. no. ABIN102010, for antibodies against Gα_i_ and β-actin) or mouse IgG (1:10000, cat. no. A4416, Sigma, for antibodies recognizing Gα_11/14/q,_ Gα_s/olf,_ Gα_12_ and Gα_13_) diluted in Roti-Block, or antibodies against mouse IgG (1:2000, cat. no. NA9310; GE Healthcare, for an antibody recognizing α-tubulin) or rabbit IgG (1:2000, cat. no. NA9340; GE Healthcare, for antibodies specific for β-arrestin1 and β-arrestin2) diluted in 5% skim milk TBS buffer.

The proteins of interest were detected by chemiluminescence using Amersham Biosciences ECL Prime Western blotting detection reagent (GE Healthcare, for antibodies recognizing rabbit IgG from ABIN and mouse IgG from Sigma) or with an in-house chemiluminescent reagent (for antibody specific for mouse IgG and rabbit IgG from GE Healthcare). To check equal loading and protein transfer, membranes were reprobed with an antibody against β-actin (1:2500, cat. no. BLD-622102 (Poly6221), BioLegend) or α-tubulin (1:200_,_ cat. no. sc-32293 (DM1A), Santa Cruz Biotechnologies).

### Peptide synthesis

All chemicals for peptide synthesis of [Sar^1^, Ile^4^, Ile^8^]AngII (SII) were purchased from Iris Biotech GmbH, Orpegen Peptide Chemicals GmbH or Merck Millipore. The peptide SII was synthesized according to a standard Fmoc (N-(9-fluorenyl)methoxycarbonyl) protocol for automated solid-phase peptide synthesis employing an EPS 221 peptide synthesizer (Intavis Bioanalytical Instruments AG). An Ile-preloaded chlorotrityl chloride resin (0.62 mmol g^−1^) was used for subsequent peptide elongation with HBTU as coupling reagent and NMM as the base. Peptide cleavage was performed at room temperature for 3 h in reagent K cleavage mixture (75 mg phenol, 25 µl ethanditiol, 50 µl thioanisol, 50 µl water, 950 µl trifluoroacetic acid per 100 mg resin). The cleavage solution was filtered and the peptide precipitated in cold diethyl ether. Crude peptide was purified by semipreparative reversed-phase HPLC using a Shimadzu LC-8A system equipped with a C18 column (Knauer Eurospher 100, 5 µm particle size, 100 Å pore size, 250 × 32 mm). Solvents for gradient elution were 0.1% TFA in water (eluent A) and 0.1% TFA in acetonitrile/water (90:10, eluent B). The detection was at 220 nm. Purity of the peptide was confirmed by analytical reversed-phase HPLC on a Shimadzu LC-10AT chromatograph equipped with a Vydac 218TP column (250 × 4.6 mm, 5 µm particle size, 300 Å pore size). SII was analyzed using a gradient from 10 to 50% eluent B (0.1% TFA in acetonitrile) in eluent A (0.1% TFA in water) in 40 min. A retention time of 19.1 min was observed for pure SII. Purity was >95% determined by HPLC. The final yield of purified peptide SII was 35%.

Identity of SII was confirmed by LC-MS analysis on an ESI (electrospray) micrOTOF-Q III system (Bruker Daltonics GmbH) connected to a Dionex Ultimate 3000 (Thermo Scientific). Samples were separated by an EC 100/2 Nucleoshell RP18 column (C18 reversed phase, 100 × 2 mm, 2.7 µm particle size, 90 Å pore size). For SII the correct molar mass (m/z 459.7981 [M+2 H]^2+^ and 306.8684 g/mol [M+3 H]^3+^) was detected (theoretical MW 917.5810 g/mol). Amino acid analysis using an Eppendorf Amino Acid Analyser LC 3000 (Eppendorf) after hydrolysis with 6 N HCl at 110 °C for 24 h revealed the expected amino acid content according to the primary sequence.

### GloSensor cAMP assay

Wild-type and ΔGsix cells were seeded in 6-cm dish at a density of 2 × 10^5^ cells ml^−1^. After 1-day culture, the cells were transfected with a pCAGGS expression plasmid (a kind gift from Dr. Jun-ichi Miyazaki at Osaka University, Japan) encoding the pGlo22F cAMP biosensor (1 µg per dish; gene synthesized with codon optimization by Genscript) together with a vasopressin V2 receptor-encoding plasmid or an empty vector (400 ng per dish) using polyethylenimine solution (10 µL of 1 mg ml^−1^ solution per dish; Polyethylenimine “Max”, (Mw 40,000); Polysciences). 24 h post transfection, cells were detached with EDTA-PBS, centrifuged and suspended in 0.01% BSA- and 5 mM HEPES (pH 7.4)-containing Hank’s Balanced Salt Solution (HBSS) (vehicle; 1 ml per dish). The cells were seeded in a half-area white 96-well plate (30 µL per well) and mixed with D-luciferin potassium solution (10 µL of 8 mM solution per well; Wako Pure Chemical, Japan). After 2 h incubation in the dark at room temperature, the plate was read for its initial luminescent count (integration time of 1 s per well; Spectramax L, Molecular Devices, Japan). The cells were treated with vehicle, 100 nM arginine vasopressin (Peptide Institutes, Japan) or 100 µM NKH-477 (Tocris) (10 µL of 5X solution per well). At 10 min, the plate was measured for its compound-treated count. The luminescent signals were normalized by the initial count and cAMP amounts were expressed as fold changes in luminescent signals.

### Bioluminescence resonance energy transfer (BRET) assays

Molecular interaction between β-arrestin2 and the receptor was measured using either GFP-tagged β-arrestin2 (acceptor) and Renilla luciferase (Rluc)-tagged receptor (donor) (for GPR17, DP2 and M3-DREADD receptors, BRET^2^) or eYFP-tagged receptor (acceptor) and Rluc-tagged β-arrestin2 (donor) (for FFA2 receptor, BRET^1^). Constructs were transfected into the indicated cell lines in a ratio of 1:4 (donor:acceptor) using fugene HD (Promega). 48 h after transfection cells were suspended in HBSS+20 mM HEPES. For BRET^1^ measurements of FFA2-eYFP-β-arrestin2-Rluc interaction 80,000 cells were seeded in 40 µl into a white 96-well plate and placed on a shaker for 30 min at 37 °C. 10 µl of Rluc substrate coelenterazin *h* (Gold Biotechnology) was applied to each well to achieve a final concentration of 5 µM. 5 min later, agonist or buffer was added to each well and placed on a shaker for 5 min and the plate was subsequently transferred to the reader for measurement. For BRET^2^ measurements 180,000 cells were seeded in 170 µl HBSS+20 mM HEPES and incubated for 30 min on a shaker at 37 °C. 10 µl 18x agonist was added and incubated for additional 10 min. Immediately after addition of 20 µl substrate DeepBlueC (Gold Biotechnology) (5 µM) BRET ratios were measured on the Mithras LB 943 multimode reader (Berthold technologies).

### DMR assay

Dynamic mass redistribution assays were conducted as described previously in detail^38^. Briefly, cells were seeded and grown overnight to confluence in 384 well EPIC biosensor plates (Corning) with 150 ng ml^−1^ pertussis toxin (PTX) where indicated. On the next day, cells were washed twice with HBSS containing 20 mM HEPES adjusted for DMSO and incubated for at least 1 h at 37 °C in the EPIC reader (Corning). FR was added 1 h before the measurement in HBSS (+HEPES) at a final concentration of 1 µM. At least 3 min of baseline read were recorded when cells were equilibrated (no change in basal DMR) and compounds were added with a liquid handling robotic (Selma, CyBio). DMR changes were monitored for at least 2 h at 37 °C. Raw data were processed using the microplate analyzer MS-Excel macro (Corning) and subsequently analyzed in GraphPad Prism. Real-time DMR records are depicted as representative experiments (mean + s.e.m.) with each trace reflecting the average of three technical replicates. Each experiment was repeated at least three times to obtain three independent biological replicates.

### HTRF-based second messenger and ERK1/2 phosphorylation assays

All homogenous time-resolved fluorescence (HTRF)-based assays were conducted according to manufacturer’s instructions (Cisbio). Briefly, for the ERK1/2 phosphorylation and total ERK1/2 assay, receptor expressing cells were seeded into 96-well poly-*D*-lysine (PDL)-treated microtiter plates and grown in complete medium overnight with 150 ng ml^−1^ pertussis toxin where indicated. Afterwards, medium was exchanged for starvation medium (growth medium without FBS) and incubated for at least 4 h at 37 °C. Cells were treated for 1 h with 1 µM FR where indicated. Cells were then stimulated with agonist for the stated times. After the supernatant was replaced by 50 µl lysis buffer the plate was incubated for 30 min at room temperature on an orbital shaker. 16 µl lysates were transferred to a white 384 well plate and incubated with 4 µl of premixed HTRF-antibody solution in the dark for at least 2 h at room temperature. HTRF ratios were measured on the Mithras LB 940 multimode reader (Berthold technologies) using emission at 665 nm and 620 nm. None of the applied stimuli affected total cellular ERK1/2 amounts (Figs. 4, 5, 7) and, therefore, total ERK1/2 was not repeatedly analyzed in Fig. 8.

For the IP (cAMP) assay, cells were suspended in stimulation buffer (Cisbio) (HBSS+20 mM HEPES+1 mM IBMX) and incubated in 7 µl (5 µl) in a 384 white microtiter plate for 20 min at 37 °C. Cells were then stimulated with 7 µl (5 µl of a forskolin/agonist mixture) and further incubated for 30 min at 37 °C. 3 µl (5 µl) of d2-antibody and 3 µl (5 µl) of cryptate-conjugated antibody were added and HTRF ratios were measured using the Mithras LB 940 multimode reader (Berthold technologies) at 665 nm and 620 nm after 1 h incubation in the dark at room temperature. cAMP measurements for GPR17 were carried out using GPR17-Rluc expressing HEK293 cells.

### ELISA assays

For ELISA assays N-terminal HA- (GPR17, β2AR, M3DREADDs) or FLAG-tagged (DP2) receptor expressing cells were seeded at a density of 50,000–60,000 cells per well in a PDL-coated clear 96-well plate and grown overnight at 37 °C and 5% CO_2_. Medium was exchanged for starvation medium (growth medium without FBS) and cells were stimulated with the indicated concentration of agonist for the stated times or left unstimulated. Medium was aspirated and cells were fixed with 4% paraformaldehyde for 20 min at room temperature. For detection of FLAG-tagged receptors all following washing and blocking solutions contained 1 mM CaCl_2_. After washing three times with PBS, cells were blocked with Blotto (3% dry milk, 50 mM TRIS-HCl, pH 7.4) for 1 h at 37 °C. Primary antibody was then incubated for 45 min at 37 °C (antibody against HA-tag 1:400, cat. no. 11583816001, Roche; antibody against FLAG M1 1:1000, cat. no. F3040, Sigma-Aldrich, in blotto). First antibody was aspirated and cells were washed three times with PBS for at least 5 min at 37 °C. Secondary antibody (antibody against mouse HRP-conjugated 1:1000, cat. no. A4416, Sigma-Aldrich, in blotto) was then incubated for 45 min in the dark at 37 °C. After washing three times with PBS for 5 min at 37 °C, 100 µl 3,3’,5,5’-Tetramethylbenzidin (TMB) solution was added to each well and incubated for 1–5 min. Reaction was stopped by adding 50 µl of 0.5 M H_2_SO_4_ to each well. Absorbance was measured at 450 nm with 620 nm reference wavelength at the TECAN sunrise absorbance reader (TECAN). For internalization rescue experiments, receptor expressing cells were transfected with β-arrestin2-GFP 24–48 h prior to measurement.

### Imaging

Microscopy was carried out on an AxioObserver inverted fluorescence microscope (Zeiss). For live cell imaging of YFP-tagged FFA2 receptors, cells were seeded onto fibronectin-coated 96-well ibidi µ-plates (ibidi) and incubated overnight at 37 °C and 5% CO_2_. Receptor expression was induced by addition of doxycycline (1 µg ml^−1^). PTX (150 ng ml^−1^) was added if stated. The next day, growth medium was exchanged for Fluorobrite DMEM (ThermoFisher) containing 10% FBS containing PTX and FR (1 µM, for 1 h) where indicated. Receptor trafficking of either vehicle- or agonist-treated cells was imaged at 37 °C for at least 30 min using the YFP-filter set. N-terminally HA-tagged GPR17 and M3D-WT receptors as well as FLAG-tagged DP2 receptors were immunostained for imaging. Receptor expressing cells were seeded onto fibronectin-coated 96-well ibidi µ-plates and grown overnight at 37 °C and 5% CO_2_. Cells were incubated with an antibody recognizing the HA-tag (1:500, cat. no. 11583816001 (12CA5), Roche) or the FLAG-tag (FLAG M1 1:1000, cat. no. F3040 (M1), Sigma-Aldrich) antibody for 30 min at 37 °C and stimulated with agonist for 30 min. Cells were then washed with PBS and fixed with 4% paraformaldehyde in PBS for 30 min at room temperature. After washing three times with PBS for 10 min at room temperature cells were permeabilized in blotto (3% milk, 0.1% triton X-100, 50 mM TRIS-HCl, pH 7.4) and stained with Cy2-conjugated anti-mouse antibody (1:500, cat. no. AP124J, Millipore) for 20 min at 37 °C. Cells were washed three times with PBS for 10 min at 37 °C and counterstained with DAPI solution (0.2 µg ml^−1^) for 15 min in the dark at room temperature. Following three washes with PBS cells were mounted and imaged in Mowiol solution. Receptors were imaged using the GFP/Cy2 filter set and nuclei were visualized using the DAPI filter set.

### FACS receptor internalization assay

Parental HEK293 cells and Δβarr1/2 cells were seeded in 6-cm dishes at cell density of 8 × 10^5^ cells per dish in 4 ml of DMEM (supplemented with 10% FBS+penicillin/streptomycin) and cultured for 1 day in a CO_2_ incubator at 37 °C. Transfection mixture was prepared by mixing 2 µg of the pCAGGS expression plasmid encoding N-terminally FLAG epitope-tagged GPCR (FLAG-V2R or FLAG-β2AR) and 10 µl of 1 mg ml^−1^ Polyethylenimine “Max” (Polysciences) in 400 µl of Opti-MEM (ThermoFisher). After 20 min incubation at room temperature, the transfection mixture was added into cells and the transfected cells were cultured for one day. Thereafter, the cells were collected by adding 300 µl of 0.53 mM EDTA-containing Dulbecco’s PBS (D-PBS), followed by 300 µl of 5 mM HEPES (pH 7.4)-containing Hank’s Balanced Salt Solution (HBSS). The cell suspension was dispensed in a 96-well V-bottom plate (200 µl per well, two wells per sample). After centrifugation at 190 g for 1 min, the cell pellets were suspended in 0.01% BSA- and 5 mM HEPES (pH 7.4)-containing HBSS (100 µl per well). Cells were mixed with 100 µl of vehicle (0.01% BSA- and 5 mM HEPES (pH 7.4)-containing HBSS) or 100 µl of 2× GPCR solution ligand (200 nM arginine vasopressin (Peptide Institute) for FLAG-V2R or 20 µM Isoproterenol (Sigma-Aldrich) for FLAG-β2AR) and incubated for 1 h in a CO_2_ incubator. After centrifugation at 1,500 g for 3 min, cells were washed once with D-PBS and centrifuged at 700 g for 1 min. The cell pellets were suspended in 2% goat serum- and 2 mM EDTA-containing D-PBS (blocking buffer; 100 µl per well) and incubated for 30 min on ice. After centrifugation at 700 g for 1 min, the cells were stained with anti-FLAG epitope tag monoclonal antibody (Clone 1E6, Wako Pure Chemicals; 10 µg ml^−1^ in blocking buffer; 50 µl per well) for 30 min on ice. After rinse with D-PBS, cells were labeled with a goat anti-mouse IgG secondary antibody conjugated with Alexa Fluor 647 (ThermoFisher Scientific; 10 µg ml^−1^ dilution in blocking buffer; 25 µl per well) for 15 min on ice. The cells were washed once with D-PBS, resuspended in 100 µl of 2 mM EDTA-containing-D-PBS and filtered through a 40 µm filter. The fluorescently labeled cells (~5,000–20,000 cells per sample) were analyzed by an EC800 flow cytometer (dual 488 nm and 642 nm laser; Sony). Fluorescent signal derived from Alexa Fluor 647 was recorded in a FL3 channel and flow cytometry data were analyzed by a FlowJo software (FlowJo). Live cells were gated with a forward scatter (FS-Peak-Lin) cutoff of 390 setting a gain value of 1.7 and samples were shown as a histogram with the FL3 channel (*s* axis). Values of mean fluorescence intensity (MFI) were used for quantification.

### Measurements of G protein activation

HEK293 cells were cultured in DMEM, 10% FBS (Biochrom), 100 U ml^−1^ penicillin G, and 100 μg ml^−1^ streptomycin sulfate at 37 °C and 7% CO_2_. For measurements of Gα_i2_ activation, HEK293 cells were seeded on 24 mm poly-D-lysin-coated microscope cover glasses 3 h before transfection. Transient transfection with 1 μg of untagged receptor (DP2, GPR17 and FFA2) and 3 μg of Gi_2_-FRET sensor (pGβ_1_-2A-yellow fluorescent protein (YFP)-Gγ_2_-IRES-Gα_i2_-mTq2 cDNA^69^) per 6-well plate and Effectene transfection reagent (Qiagen) was performed according to manufacturer’s instructions. FRET measurements were accomplished 48 h after transfection. The Gq-FRET sensor^36^ was transfected as described above with a DNA ratio of 3:1 (FRET sensor: receptor). During the experiment, cells were superfused with measuring buffer (140 mM NaCl, 5.4 mM KCl, 2 mM CaCl_2_, 1 mM MgCl_2_, 10 mM HEPES, pH 7.3) supplemented with the respective ligand (PGD_2_ 1 µM, MDL 10 µM, and C3 1 mM) at indicated time points, using a pressure-controlled perfusion system (ALA Scientific).

FRET measurements were carried out on an inverted microscope (Zeiss Axiovert 200) equipped with an oil immersion 63 × objective lens and a dual-emission photometric system (Till Photonics). The transfected cells were excited with light from a polychrome IV (Till Photonics) at a frequency of 10 Hz with 20 ms illumination out of a total time of 100 ms. Emission of cyan fluorescent protein (CFP, 480 ± 20 nm) and YFP (535 ± 15 nm), and the FRET ratio (FYFP/FCFP) were monitored simultaneously (beam splitter DCLP 505 nm) upon excitation at 436 ± 10 nm (beam splitter DCLP 460 nm). Fluorescence signals were detected by photodiodes, digitalized using an analog-digital converter (Digidata 1440 A, Axon Instruments) and recorded with Clampex 9.0 software (Science Products). For data analysis OriginPro 9 was used.

### Statistical analyses

Statistical analyses were performed in Prism software 6.01 (GraphPad), and specific tests are noted in the figure legends. Data sets with normal distributions were analyzed using two-tailed, paired or unpaired Student’s *t*-tests and presented as means + s.e.m. (Fig. 1 presented as means + s.d.), if not otherwise indicated. Comparisons with normalized data (control group set to 100) were analyzed by one-sample, two-sided *t*-test. Two-sample paired Wilcoxon test was applied to paired points at different times or concentrations, with a confidence-level of 95%. Sample size was chosen to allow sufficient statistical power. *P*-values are indicated as follows: *P < 0.05; **P < 0.01; ***P < 0.001.

### Data availability

The authors declare that all data supporting the findings of this study are available within the article and its Supplementary Information files, and from the corresponding author upon reasonable request.

## Electronic supplementary material

Supplementary Information

Peer Review File
