# Supplementary material for: Lack of beta-arrestin signaling in the absence of active G proteins
Source: Nat Commun. 2018 Jan 23;9:341. doi: 10.1038/s41467-017-02661-3 (PMC5780443; doi:10.1038/s41467-017-02661-3)
Supplement: Supplementary file 1 — Supplementary Information [file 41467_2017_2661_MOESM1_ESM.pdf]

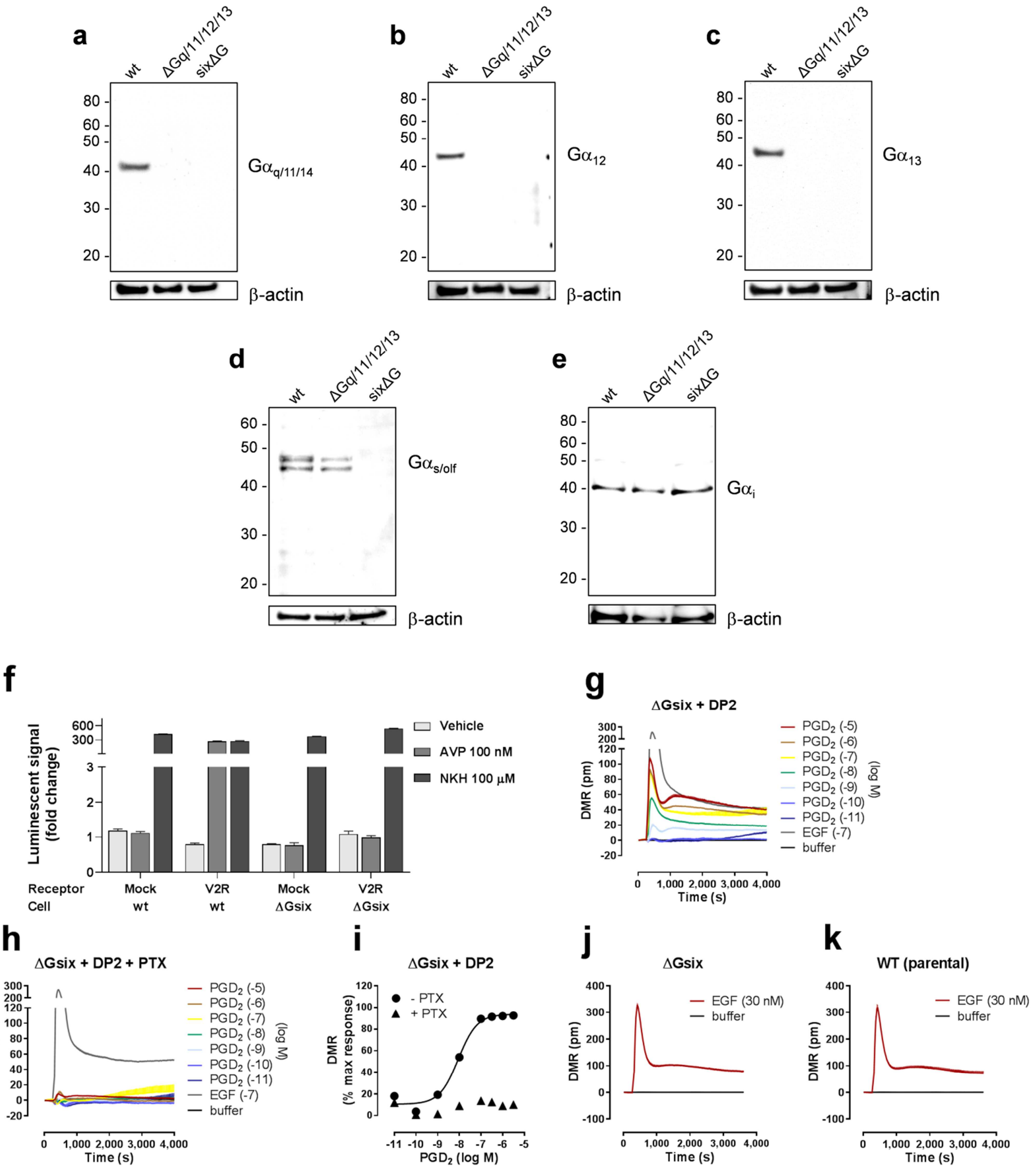

Supplementary Figure 1. Biochemical and functional characterization of ΔGsix cells. (**a-e**) Western blots of wild-type, ΔGq/11/12/13 and ΔGsix HEK293 cell lysates probed with antibodies against $G\alpha_{q/11/14}$ (**a**), $G\alpha_{12}$ (**b**), $G\alpha_{13}$ (**c**), $G\alpha_{s/olf}$ (**d**) and $G\alpha_{i}$ (**e**). Uncropped blots are available in Supplementary Fig. 17. (**f**) cAMP levels after stimulation of vasopressin V2 receptor (V2R) or mock transfected wild-type and ΔGsix cells with the indicated compounds or vehicle for 10 minutes. (**g,h**) DMR traces of PGD_2-stimulated DP2 receptor in ΔGsix HEK293 cells in absence (**g**) and presence (**h**) of PTX. (**i**) Concentration-response-curve of peak value data from (**g**) and (**h**). (**j,k**) Real-time whole cell response of ΔGsix (**j**) and wild-type (**k**) HEK293 cells upon stimulation with 30 nM epidermal growth factor (EGF). (**a-e**) Representative blots of 2 independent experiments. (**f,i**) Data are mean +/± SEM of 3 independent experiments. (**g,hj,k**) Shown are representative traces (mean + SEM) of 3 independent experiments, each performed in triplicate.

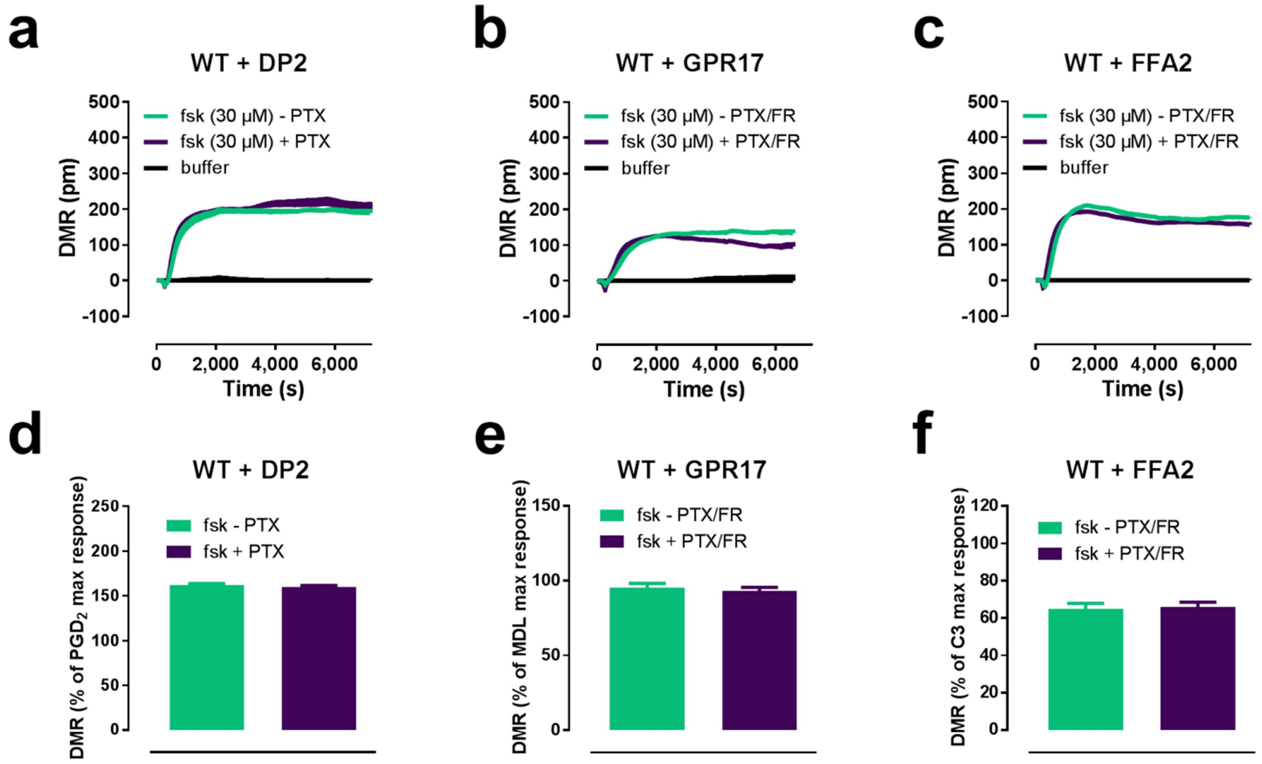

Supplementary Figure 2. Forskolin-mediated DMR in the absence and presence of signal transduction inhibitors PTX and FR. (**a-c**) Forskolin (fsk)-induced DMR traces of DP2 (**a**), GPR17 (**b**), or FFA2 (**c**) wild-type HEK293 cells in absence and presence of G protein inhibitors. (**d-f**) Quantification of fsk-mediated DMR responses from conditions in (**a-c**). (**a-c**) Shown are representative traces (mean + SEM) of 3 independent experiments, performed in triplicate wells for each condition. (**d-f**) AUC of DMR traces over the entire measurement time was analyzed and presented as mean values + SEM of 3 independent experiments (3 technical replicates).

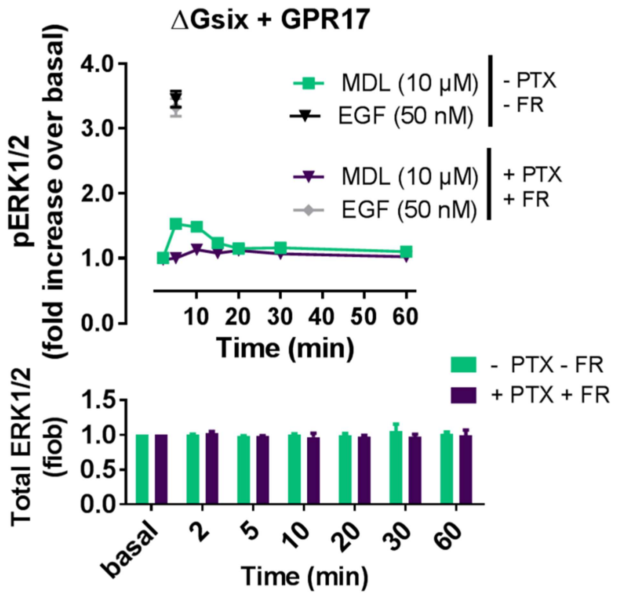

Supplementary Figure 3. G protein-dependence of ERK1/2 phosphorylation for the GPR17 receptor. 10 µM MDL-induced kinetic pERK1/2 and total ERK profile of GPR17 in ΔGsix cells (+/- PTX/FR). Data are mean ±/+ SEM of 3 independent experiments, each measured in triplicates. Fiob, fold increase over basal.

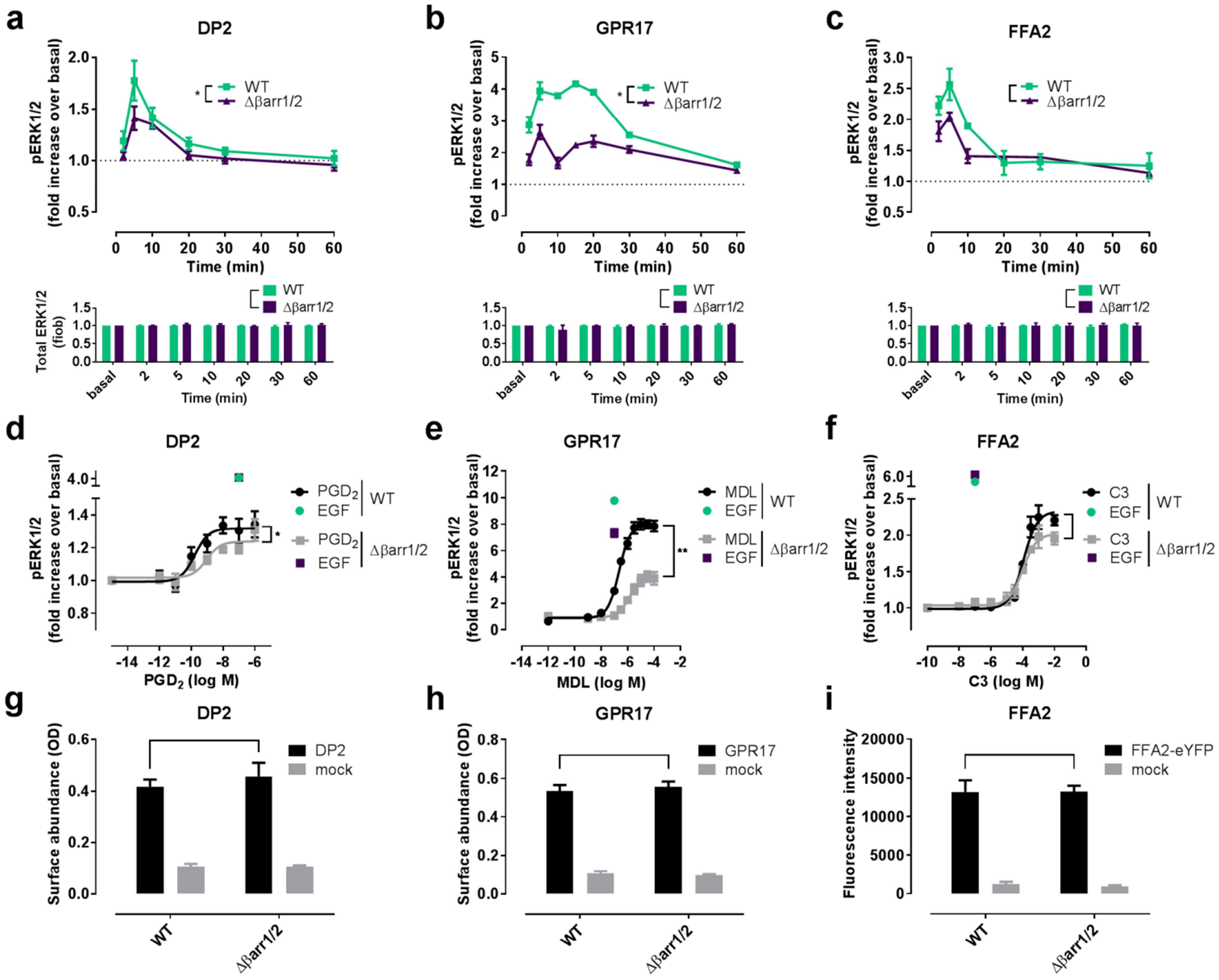

Supplementary Figure 4. Comparative analysis of ERK1/2 phosphorylation and cell surface abundance of DP2, GPR17 and FFA2 in parental HEK293 and Δβarr1/2 cells. (**a-c**) pERK1/2 and total ERK1/2 kinetic profile of DP2 (**a**), GPR17 (**b**), and FFA2 (**c**) in wild-type and Δβarr1/2 cells stimulated with 1 µM PGD_2 (**a**), 10 µM MDL (**b**), and 100 µM C3 (**c**). (**d-f**) Concentration-effect-curves of pERK1/2 levels after 5 minutes stimulation of WT and Δβarr1/2 cells expressing DP2 (**d**, pEC_{50}: 9.78±0.32 (WT); 9.0±0.35 (Δβarr1/2)), GPR17 (**e**, pEC_{50}: 6.64±0.7 (WT); 5.86±0.14 (Δβarr1/2)) or FFA2 (**f**, pEC_{50}: 4.0±0.1 (WT); 4.0±0.12 (Δβarr1/2)) with their cognate agonist. (**g-i**) Quantification of receptor expression in wild-type and Δβarr1/2 cells of DP2 (**g**), GPR17 (**h**), and FFA2eYFP (**i**). Data are mean +/± SEM of 3 independent experiments (3 technical replicates). For statistical analysis, two-sample paired Wilcoxon test was applied to paired points at different times (**a-c**) or at different concentrations (**d-f**) and two-tailed paired t-test (**g-i**) were performed. *, P<0.05; **, P<0.01; not significant where no asterisk. Fiob, fold increase over basal.

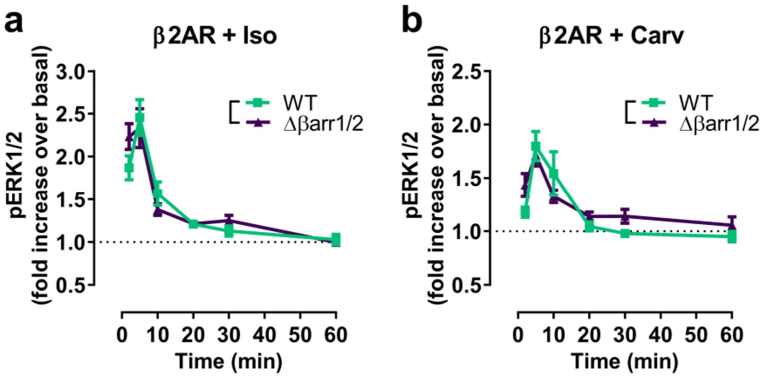

Supplementary Figure 5. Comparative analysis of ERK1/2 phosphorylation for ligand-stimulated β2AR in parental HEK293 and Δβarr1/2 cells. (**a**,**b**) pERK1/2 kinetic profiles of β2AR in wild-type parental and Δβarr1/2 HEK293 cells stimulated with 10 µM isoproterenol (Iso) (**a**) and 10 µM carvedilol (Carv) (**b**). Data are mean ± SEM of 4 independent experiments (3 technical replicates each). For statistical analysis, two-sample paired Wilcoxon test was applied to paired points at different times; not significant where no asterisk.

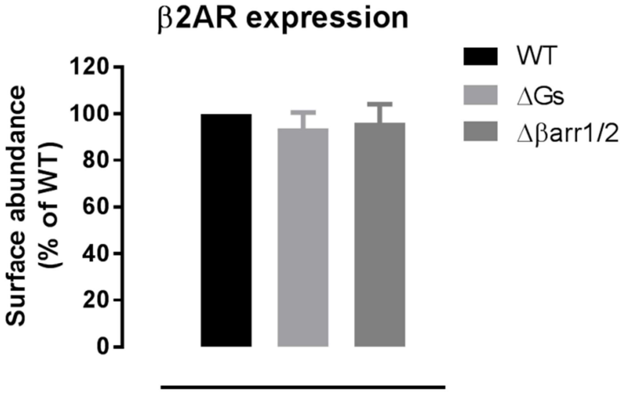

Supplementary Figure 6. Cell surface abundance of β2-adrenergic receptor (β2AR) in different HEK293 cell lines. Surface ELISA detecting N-terminally HA-tagged β2AR in wild-type, ΔGs and Δβarr1/2 HEK293 cells. Data are mean + SEM of 3 independent experiments, performed in triplicate.

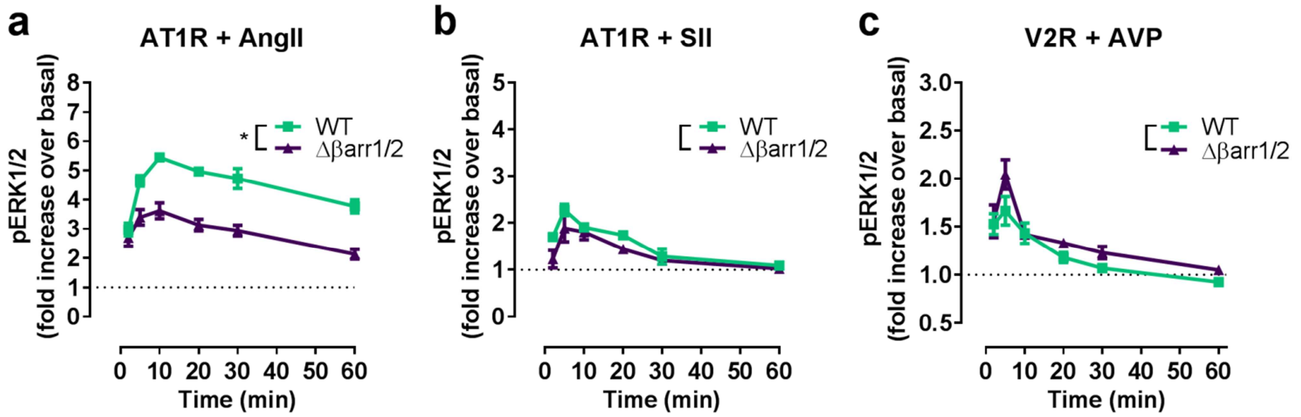

Supplementary Figure 7. Comparative analysis of ERK1/2 phosphorylation for ligand-stimulated AT1R and V2R in parental HEK293 and Δβarr1/2 cells. (**a-c**) pERK1/2 kinetic profiles of AT1R (**a,b**) and V2R (**c**) in wild-type parental and Δβarr1/2 HEK293 cells stimulated with 100 nM angiotensin II (AngII) (**a**), 30 μM [Sar^1, Ile^4, Ile^8]AngII (SII) (**b**), and 1 μM arginine-vasopressin (AVP) (**c**). Data are mean ± SEM of 4 (AT1R in WT), 5 (AT1R in Δβarr1/2), and 3 (V2R) independent experiments (3 technical replicates each). For statistical analysis, two-sample paired Wilcoxon test was applied to paired points at different times. *, $P<0.05$; not significant where no asterisk.

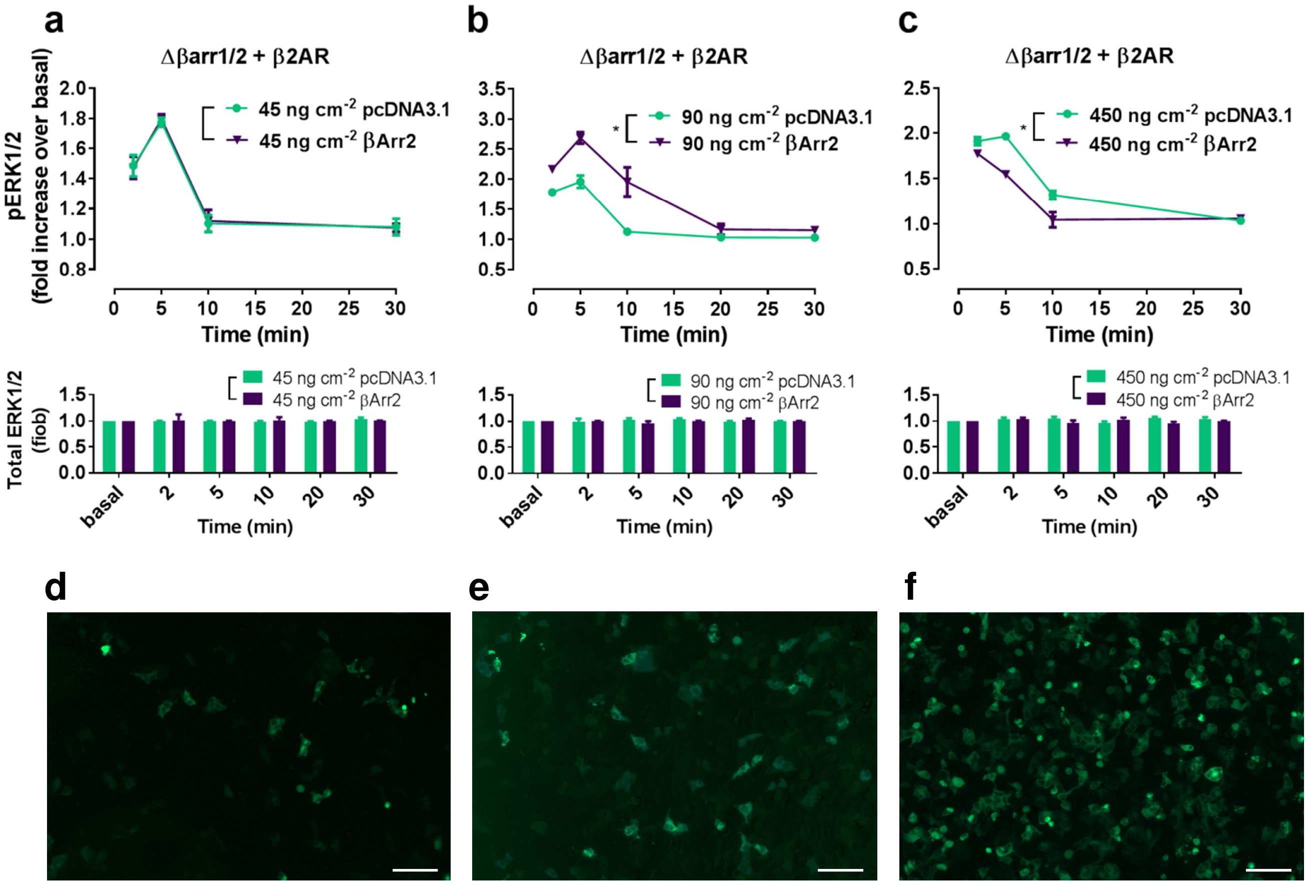

Supplementary Figure 8. Impact of increasing β-arrestin2 amounts on β2AR-mediated pERK1/2 kinetics. (**a-c**) Kinetic ERK1/2 phosphorylation and total ERK profile of 10 µM Isoproterenol-stimulated Δβarr1/2 cells stably transfected with β2AR and transiently transfected with either empty vector (pcDNA3.1) or a range of β-arrestin2-GFP (βArr2) amounts. (**d-f**) Fluorescence imaging of β-arrestin2 transfected cells for each condition in a-c. (**a-c**) Data are mean ± SEM of 4 independent experiments (3 technical replicates). For statistical analysis, two-sample paired Wilcoxon test was applied to paired points at different times. *, P<0.05; not significant where no asterisk. (**d-f**) Representative images of 4 independent experiments. Scale bar is 100 µm.

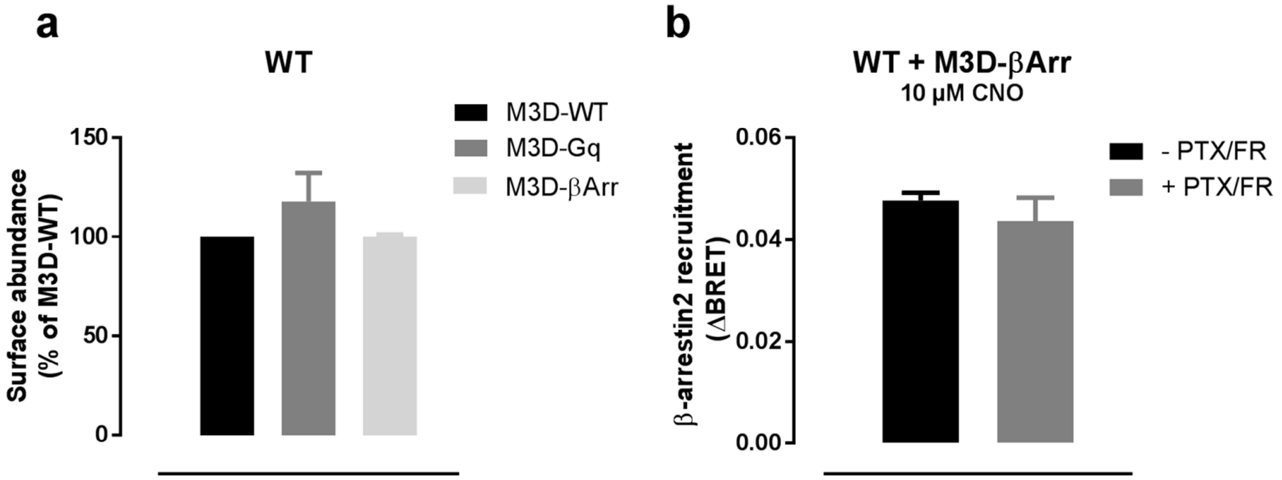

Supplementary Figure 9. Cell surface abundance and functionality of M3D-βArr in wild-type HEK293 cells. (**a**) Surface amounts of N-terminally HA-tagged DREADD constructs (M3D-WT, M3D-Gq, M3D-βArr) in wild-type (WT) HEK293 cells. (**b**) CNO-induced β-arrestin2 recruitment of the M3D-βArr receptor in absence (black) and presence (grey) of G protein inhibitors (PTX/FR). Data are mean + SEM of 3 independent experiments, each measured in triplicates.

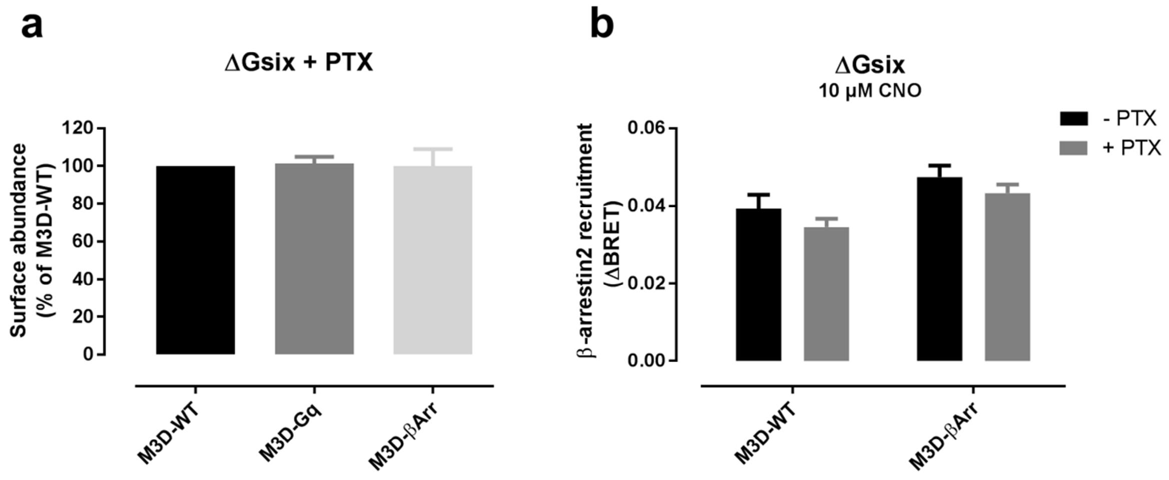

Supplementary Figure 10. Cell surface DREADD abundance and β-arrestin2 recruitment in the absence of G proteins. (**a**), Surface ELISA of N-terminally HA-tagged DREADD constructs (M3D-WT, M3D-Gq, M3D-βArr) in HEK293 cells collectively lacking G proteins (ΔGsix + PTX). (**b**) β-arrestin2 recruitment by M3D-WT and M3D-βArr receptors upon CNO stimulation in cells either partially (ΔGsix, black) or collectively (ΔGsix + PTX, grey) depleted of G proteins. Data are mean + SEM of 3 independent experiments (3 technical replicates).

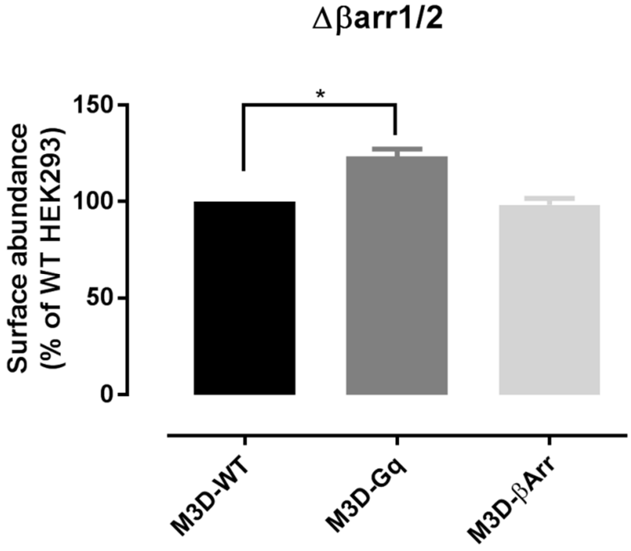

Supplementary Figure 11. DREADD cell surface abundance in Δβarr1/2 cells. Surface ELISA of N-terminally HA-tagged DREADD constructs (M3D-WT, M3D-Gq, M3D-βArr) in Δβarr1/2 cells, normalized to surface abundance of receptors in WT HEK293 cells (100%). Data are mean + SEM of 3 independent experiments, performed in triplicate. For statistical analysis, one sample t-test was performed. *, $P<0.05$.

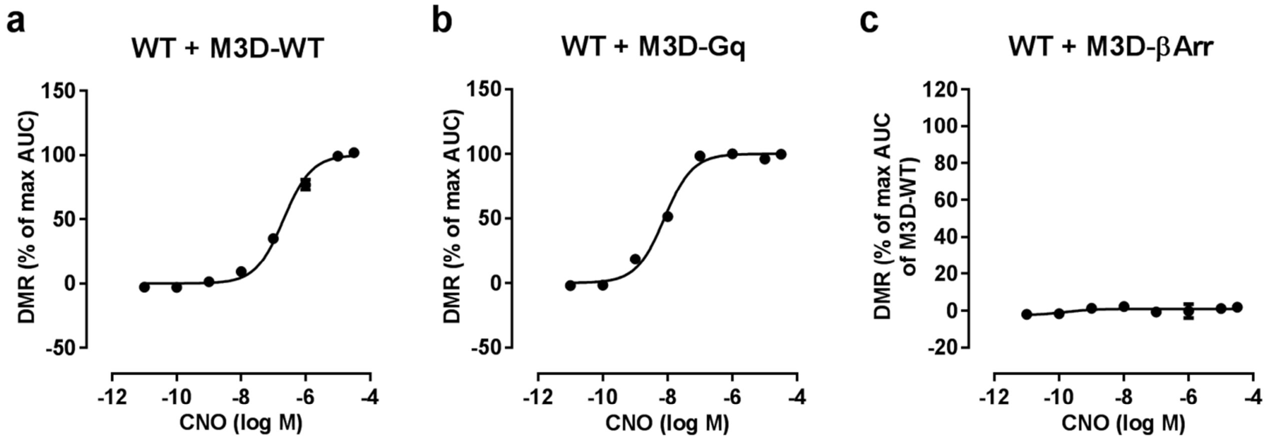

Supplementary Figure 12. Concentration-effect curves of DMR recordings for CNO-activated DREADDs in HEK293 cells. Quantification of CNO-induced DMR response in wild-type HEK293 cells expressing the M3D-WT (pEC_{50}: 6.68) (**a**), the M3D-Gq (pEC_{50}: 8.12) (**b**), or the M3D-βArr (**c**) receptor. AUC of DMR traces over the entire measurement time was analyzed and presented as mean values ± SEM of 3 independent experiments (3 technical replicates).

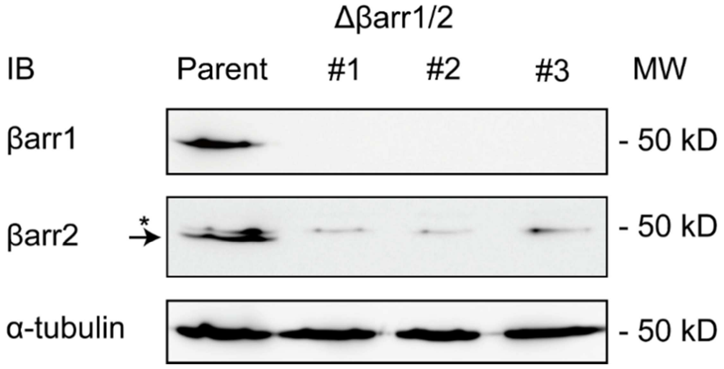

Supplementary Figure 13. Biochemical characterization of Δβarr1/2 HEK293 cell lines using immunoblotting. Western blots of lysates derived from wild-type (Parent) and three Δβarr1/2 HEK293 cell clones (#1, #2, #3) probed with antibodies against β-arrestin1 (βarr1) and β-arrestin2 (βarr2). Antibody against β-arrestin2 shows a minor non-specific band (asterisk). Western blot images are representative of two independent experiments with each showing similar patterns of immune-reactive bands. Unprocessed scans of Western blots are available in Supplementary Fig. 18.

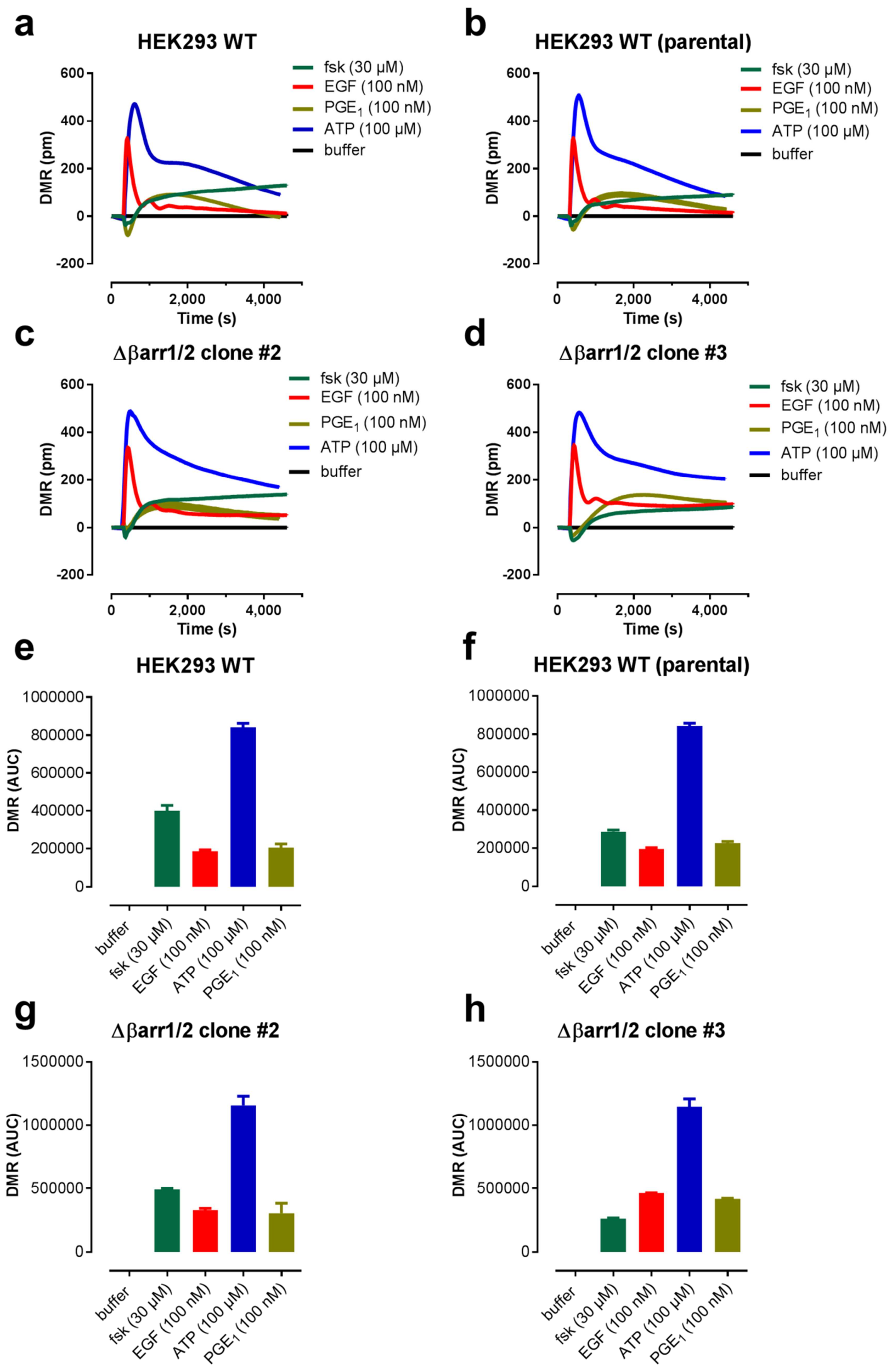

Supplementary Figure 14. DMR analysis of different HEK293 cell clones. Quantification of DMR response towards multiple stimuli in wild-type (**a**), wild-type (parental) (**b**), Δβarr1/2 clone #2 (**c**) and Δβarr1/2 clone #3 (**d**). (**e-h**) AUC of DMR traces over the entire measurement time was analyzed and presented as mean values + SEM of 3 independent experiments, each measured in triplicates.

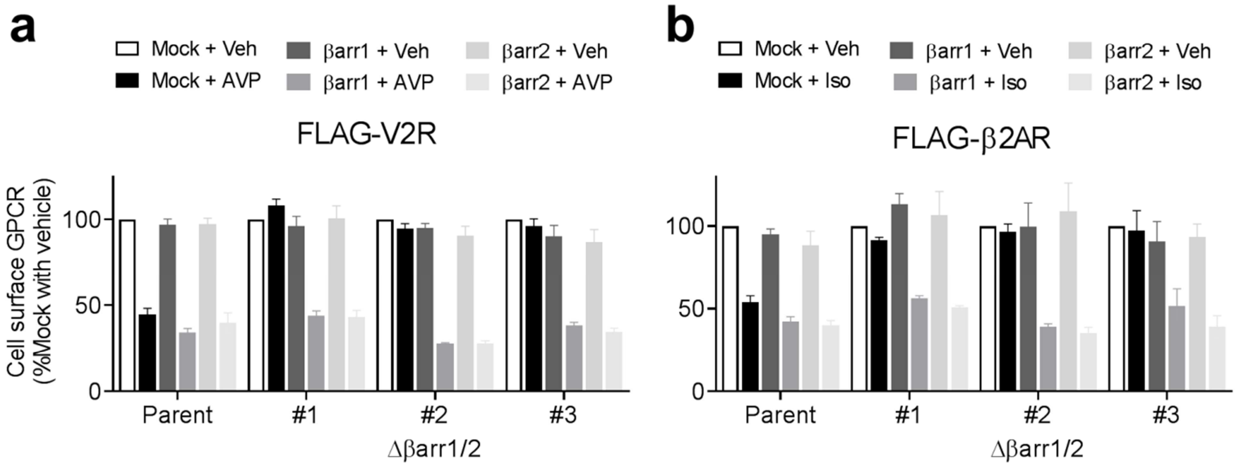

Supplementary Figure 15. Agonist-mediated internalization in wild-type (parental) and Δβarr1/2 HEK293 clones #1, #2, and #3. Quantification of cell surface receptor abundance of FLAG-tagged Vasopressin V2 receptor (FLAG-V2R) (**a**) and FLAG-tagged β2-adrenergic receptor (FLAG-β2AR) (**b**) in parental HEK293 cells (Parent) and Δβarr1/2 clones #1, #2, and #3, either transfected with vector (Mock) or β-arrestin1 (βarr1) or β-arrestin2 (βarr2) and stimulated with 100 nM AVP (**a**) or 10 µM Isoproterenol (Iso) (**b**). Data are mean + SEM of 4 independent experiments (3 technical replicates).

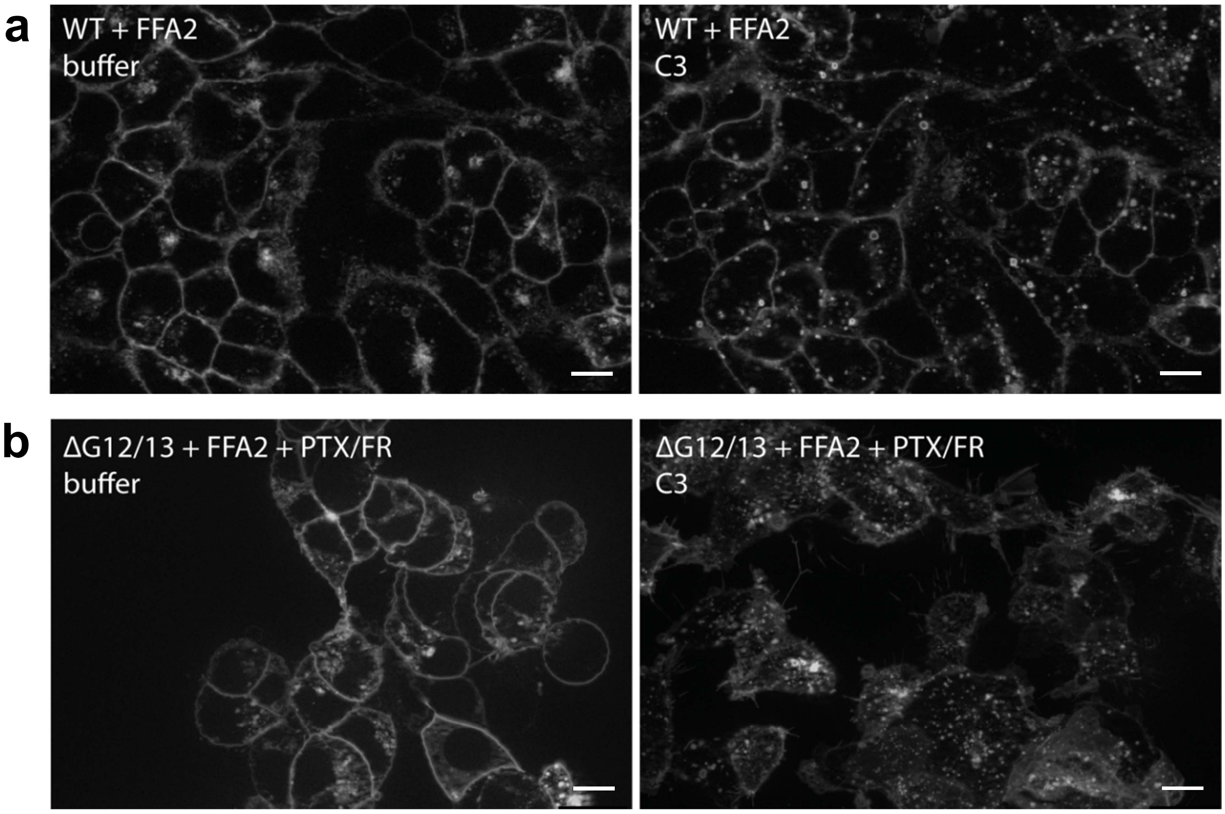

Supplementary Figure 16. FFA2 receptor internalization in the absence of active G proteins. Structured-illumination micrographs of YFP-tagged FFA2 receptors in wild-type (**a**) or ΔG12/13 (**b**) cells, imaged 30 minutes after buffer treatment (left panels) or stimulation with 100 µM C3 (right panels). Scale represents 10 µm; representative images of 3 independent experiments.

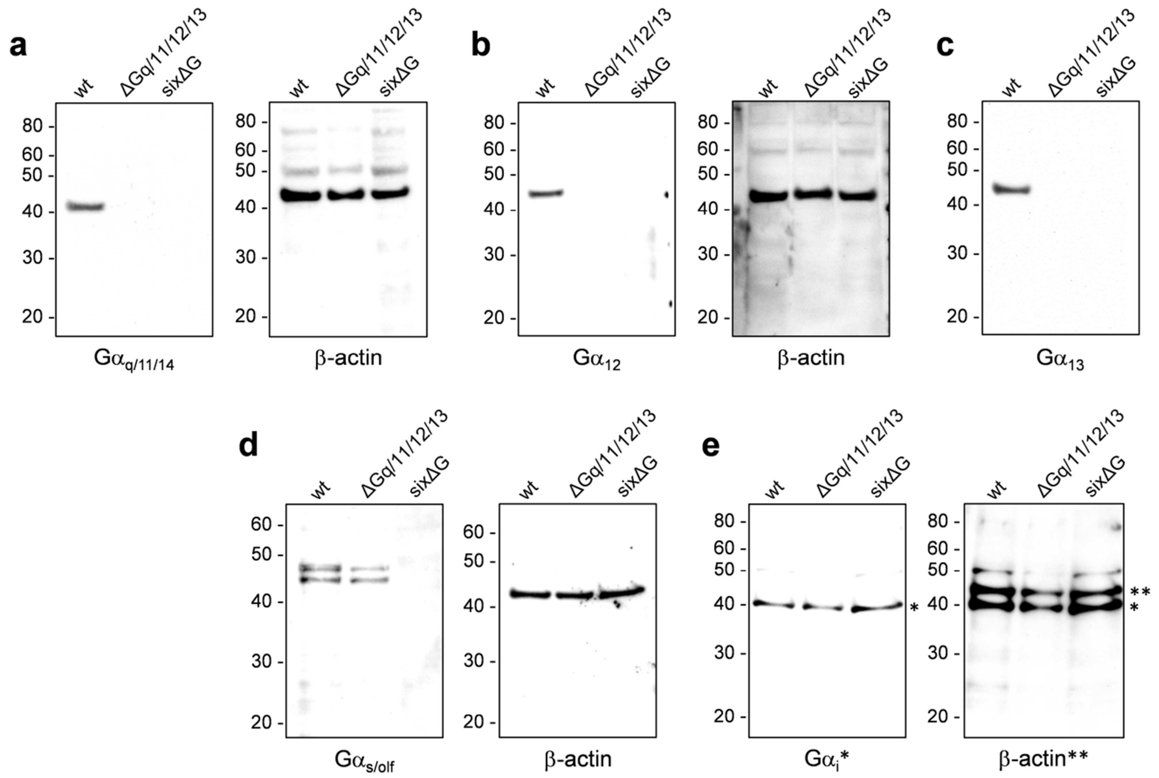

Supplementary Figure 17. Biochemical characterization of ΔGsix cells by immunoblot analysis. (**a-e**) Uncropped images of Western blots shown in Supplementary Fig. 1**a-e** with wild-type, ΔGq/11/12/13 and ΔGsix HEK293 cell lysates probed against $G\alpha_{q/11/14}$ (**a**), $G\alpha_{12}$ (**b**), $G\alpha_{13}$ (**c**), $G\alpha_{s/olf}$ (**d**), $G\alpha_i$ (**e**), and β-actin (**a-e**). (**e**) Note that $G\alpha_i$ is also visible on the β-actin blot because $G\alpha_i$ and β-actin antibody were from the same host species and corresponding immunoreactive bands were detected with similar illumination time (*: $G\alpha_i$, **: β-actin). Representative blots of 2 independent experiments.

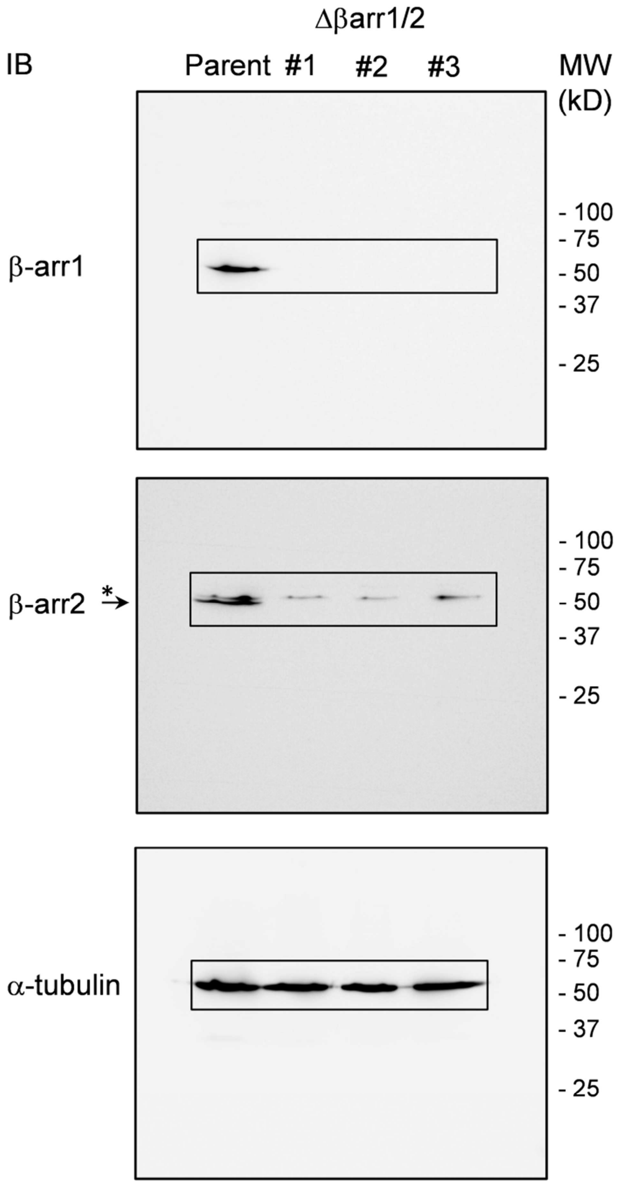

Supplementary Figure 18. Biochemical characterization of Δβarr1/2 HEK293 cell lines using immunoblotting. Unprocessed scans of Western blots presented in Supplementary Fig. 13 with lysates derived from wild-type (Parent) and three Δβarr1/2 HEK293 cell clones (#1, #2, #3) probed against β-arrestin1 (βarr1), β-arrestin2 (βarr2) and α-tubulin. Antibody against β-arrestin2 shows a minor non-specific band (asterisk). Cropped images are boxed. Blots are representative of 2 independent experiments.
